# Non-Apoptotic Programmed Cell Death-Related Gene Signature Correlates With Stemness and Immune Status and Predicts the Responsiveness of Transarterial Chemoembolization in Hepatocellular Carcinoma

**DOI:** 10.3389/fcell.2022.844013

**Published:** 2022-04-29

**Authors:** Guixiong Zhang, Wenzhe Fan, Hongyu Wang, Jie Wen, Jizhou Tan, Miao Xue, Jiaping Li

**Affiliations:** ^1^ Department of Interventional Oncology, The First Affiliated Hospital of Sun Yat-Sen University, Guangzhou, China

**Keywords:** hepatocellular carcinoma, programmed cell death, gene signature, TP53 mutation, stemness, tumor microenvironment, transarterial chemoembolization

## Abstract

**Background:** Non-apoptotic programmed cell death, including autophagy, ferroptosis, and pyroptosis, newly discovered in recent years, plays an important role in hepatocellular carcinoma (HCC). So, this study attempted to explore the relationship between non-apoptotic programmed cell death-related genes and the molecular characteristics, tumor microenvironment, and prognosis in HCC patients.

**Methods:** The transcriptomic and clinical data of HCC samples were downloaded from various public datasets, followed by acquiring non-apoptotic programmed cell death-related genes from the database. A gene signature model was then constructed using univariate and multivariate Cox regression analyses and validated in other cohorts as well as our institution sequencing data. Kaplan–Meier survival curves and time-dependent receiver operating characteristic curves were generated to evaluate the model’s predictive capability. Furthermore, the relationships among the gene signature, TP53 mutation, stemness, immune status, and responsiveness of transarterial chemoembolization (TACE) were analyzed.

**Results:** The gene signature model was constructed based on five autophagy-, three ferroptosis-, and two pyroptosis-related differentially expressed genes. The model accurately predicted that patients classified as low risk would have better overall survival than high-risk patients, which was robustly consistent with data from other cohorts as well as our institution sequencing data. The comprehensive results indicated that a high-risk index was correlated with a high TP53 mutation rate, high cancer cell stemness, high infiltration of immunosuppressive cells and low immunophenoscore, and low TACE responsiveness of HCC patients.

**Conclusion:** Collectively, the established non-apoptotic programmed cell death-related gene signature was shown to accurately predict prognosis, associated with the TP53 mutation and liver cancer cell stemness, reflect the tumor immune microenvironment, and predict TACE responsiveness in HCC patients.

## Introduction

In 2020, liver cancer represented the sixth most common cancer type worldwide, accounting for 4.5% of all new tumors and the third leading cause of cancer-related death (over 8.3%) (Sung et al., 2021). Among the primary types of liver cancer, hepatocellular carcinoma (HCC) is the most common, accounting for approximately 75–85% of all liver cancer cases. Even patients who are diagnosed early and undergo radical liver resection still experience a high recurrence rate (Famularo et al., 2018; Fujiwara et al., 2018). Transarterial chemoembolization (TACE) is the preferred first-line regimen for patients with unresectable local lesions and good liver function, which is also the most widely applied treatment regimen not only in intermediate stage HCC recommended by guidelines but also in advanced stages according to the BRIDGE study (Park et al., 2015; Sieghart et al., 2015; European Association for the Study of the Liver, 2018; Chang et al., 2020). Due to the high recurrence of HCC even after surgical resection, postoperative adjuvant TACE is commonly used (Kudo 2011; Wang et al., 2018). Although objective tumor response rates after transarterial chemoembolization are generally favorable and may exceed 60%, more than 40% of patients have no response to TACE therapy (Gaba et al., 2015; Martin et al., 2020). Recently, immunotherapy has gradually become a hot spot in the treatment of liver cancer, whose characteristic is to stimulate a specific immune response, following inhibiting and killing tumor cells, so as to reduce the recurrence rate and metastasis rate of tumor (Inarrairaegui et al., 2018; Llovet et al., 2018). Due to the complex etiology and high heterogeneity of hepatocellular carcinoma, prognosis and treatments for HCC remain unsatisfactory despite these currently available therapies in a real-world scenario (Cancer Genome Atlas Research Network, 2017). To overcome this, there is an additional need for the development of novel prognostic models to tailor more personalized treatment regimens resulting in improving treatment outcomes in patients with HCC.

Programmed cell death (PCD) plays a fundamental role in animal development and tissue homeostasis. Abnormal regulation of this process is associated with a wide variety of human diseases, including immunological and developmental disorders, neurodegeneration, and cancer (Fuchs and Steller, 2011). Three new mechanisms of PCD, including autophagy, ferroptosis, and pyroptosis, which are different from traditional apoptosis and play a double-edged sword role in the development and progression of tumors under the regulation of related genes, were recently discovered and collectively play critical roles in HCC tumorigenesis and progression (Mishra et al., 2018; Fritsch et al., 2019; Tang et al., 2019; Bedoui et al., 2020). Autophagy, or type II programmed cell death, is a process in which HCC tumor cells may utilize it as a protective mechanism in order to adapt to external stress, thereby enhancing the tumor’s proliferation, metastasis, and resistance to treatment under the regulation of autophagy-related genes (Toshima et al., 2014; Yu et al., 2017; Levine and Kroemer 2019). Ferroptosis is an iron-dependent form of programmed cell death that has been confirmed that ferroptosis affects the development of HCC through the GPX4, P53, and MT1G signaling pathways and related genes (Dixon et al., 2012; Nie et al., 2018; Tang et al., 2021). Pyroptosis, also known as cell inflammatory death, is mediated by intracellular inflammasomes and caspase-1, which may also affect the development of HCC under the regulation of related genes (Xia et al., 2019; Al Mamun et al., 2020; Fang et al., 2020). Several studies have shown that autophagy- or ferroptosis- or pyroptosis-related gene signatures can potentially predict the prognosis of HCC or other tumors (Huo et al., 2020; Liang et al., 2020; Ju et al., 2021; Ye et al., 2021). While prognostic models have been constructed independently for each PCD-related group of genes, a prognostic model integrating all three non-apoptotic programmed cell death-related genes has yet to be developed.

In this study, we used the gene expression profile of public datasets to find three non-apoptotic programmed cell death genes related to the prognosis of HCC and then used these genes to construct a prognostic model of HCC to predict the survival of HCC patients. While first tested in other public cohorts, this prognostic model was then also verified in our cohort. Meanwhile, we also explored the relationships among gene signature, TP53 mutation, liver cancer cell stemness, and immune status, as well as the responsiveness of TACE in HCC. In the present work, it is demonstrated that an alternative prognostic model is tightly correlated with TP53 mutation, liver cancer cell stemness, and immune status, as well as TACE responsiveness and the prognosis of HCC patients.

## Materials and Methods

### Patients and Datasets

Data on HCC patients were accessed from The Cancer Genome Atlas (TCGA), International Cancer Genome Consortium (ICGC), and Gene Expression Omnibus (GEO). The transcriptome data of 374 hepatocellular carcinoma (HCC) and 50 normal samples were obtained from TCGA to analyze expression differences of the autophagy-, ferroptosis-, and pyroptosis-related genes. Inclusion criteria of patients in follow-up analysis were based on the following: 1) HCC diagnosis confirmed via pathology; 2) available RNA expression data; and 3) complete clinical data and follow-up time.

A list of 232 autophagy-, 60 ferroptosis-, and 40 pyroptosis-related genes were accessed from the HADB database (http://www.autophagy.lu/), FerrDb database (http://www.zhounan.org/ferrdb/), and the pyroptosis gene sets in the molecular signature database (MSigDB, https://www.gsea-msigdb.org/gsea/msigdb/), respectively. Other previously published literature was also used.

### Construction and Validation of the Prognostic Gene Signature

The R package “limma” in R software was used to identify the differentially expressed genes (DEGs) between tumor tissues and adjacent non-tumorous tissues, according to the criteria of | log 2 (Fold Change) | > 1 and a false discovery rate (FDR) < 0.05. Next, autophagy-, ferroptosis-, and pyroptosis-related DEGs were extracted from all DEGs. The association between autophagy-, ferroptosis-, and pyroptosis-related DEGs and patient survival was evaluated by univariate Cox regression analysis using the R package “survival.” Autophagy-related DEGs with a *p* < 0.001, ferroptosis-related DEGs with a *p* < 0.01, and pyroptosis-related DEGs with a *p* < 0.05 were considered candidate variables in a univariate Cox regression analysis. An interaction network for the prognostic DEGs was generated by the STRING database (https://www.string-db.org/) and entered into a stepwise multivariate Cox regression analysis to identify covariates with independent prognostic values for patient survival. Each patient was assigned a risk index (RI) based on the expression of predictive genes and the multivariate COX regression risk model coefficients. The risk score was calculated as follows: risk score = $\overset{n}{\underset{i=1}{\sum }}\left(GeneExpressioni\times Coefi\right)$ . In order to standardize and normalize the risk score, the risk index was introduced and calculated as follows: risk index = (risk score-min)/(max-min).

Based on the median risk index (RI), HCC patients were divided into high- and low-risk groups. The survival analysis was analyzed via Kaplan–Meier (K–M) methods to compare the high- and low-risk groups according to predictive signatures. In addition, the predictive value of prognostic prediction models was evaluated by areas under the curve (AUC) of the receiver operator characteristic (ROC) curve using the R package “survivalROC.” The principal component analysis (PCA) was performed to examine the clustering efficacy of the selected signatures with the “prcomp” function of the R package “stats.” The ICGC cohort was used to verify these results.

### Association Between the Risk Model and Clinicopathological Factors and Construction of a Prognostic Nomogram

Univariate and multivariate analyses of clinical pathology were performed with Cox regression and then used as an independent predictive risk factor for overall survival in the TCGA cohort. In order to quantitatively predict the survival risk for HCC patients, the nomogram was further constructed on the basis of the risk index as well as clinical parameters. The calibration curve was used to evaluate the accuracy of the nomogram. The nomogram and calibration curves were both plotted via R package “rms.”

### Comprehensive Analysis of Molecular Characteristics in Different Subgroups

For TP53 mutation analysis, the information on genetic alterations was obtained from TCGA and ICGC, and the quantity of TP53 mutation and tumor mutation burden (TMB) was calculated by using the R package “Maftools.” Based on the reported “YAMASHITA_LIVER_CANCER_STEM_CELL_UP and DN” gene set (Yamashita et al., 2009) (the LCSCs_UP gene set includes genes upregulated in liver CSCs, which is positively correlated with stemness phenotype of HCC, whereas the LCSCs_DN gene set is the opposite), the liver cancer stemness score of HCC samples was further inferred from transcriptomes using single-sample Gene Set Enrichment Analysis (ssGSEA) by the R package “gsva.” The stemness score was calculated as follows: stemness score = Score^STEM_CELL_UP^ + (1 - Score^STEM_CELL_DN^).

### Assessment of the Tumor Immune Microenvironment

Immune cell infiltration was estimated from RNA-sequencing data using xCell (https://xcell.ucsf.edu/, Charoentong or Rooney), which is an excellent online tool for analyzing the expression matrix of immune cell subtypes based on the principle of deconvolution algorithm. The infiltrating score of 16 immune cells and the activity of 13 immune-related pathways were further calculated with single-sample Gene Set Enrichment Analysis (ssGSEA) by the R package “gsva.” The immunophenoscore (IPS) score can well predict the response of immune checkpoint inhibitors (ICIs), which are derived from The Cancer Immunome Atlas (TCIA) (https://tcia.at/home) and calculated based on the expression of important components of tumor immunity.

### The Relationship Between the Gene Signature and Transarterial Chemoembolization Treatment

In order to analyze the relationship between the gene signature and TACE treatment, GSE104580, a comparative analysis of gene expression datasets of TACE responders and non-responders among HCC patients, was obtained from GEO. The HCC patients of GSE104580 were divided into high- and low-risk groups according to the median risk index, and then, the TACE response of the subgroups was compared. The survival analysis of 96 HCC patients treated with TACE (including a list of 69 adjuvant TACE and 27 post-recurrence TACE) in GSE14520 was estimated to compare the high- and low-risk groups according to predictive signatures.

### Verifying the Relationship Between the Gene Signature and TP53 Mutation, Stemness, Immune Status, and Transarterial Chemoembolization Treatment in Our Institution

The transcriptome sequencing data of 12 HCC patients (collected tumor samples were sent to MyGene Diagnostics (Guangzhou) for targeted next-generation sequencing analysis), who were diagnosed with HCC from 2015 to 2020 and admitted to The First Affiliated Hospital of Sun Yat-sen University in Guangzhou, China, were obtained to analyze the relationships among the gene signature, survival, TP53 mutation, stemness, immune status, and TACE treatment. TACE procedures were performed by two experienced interventional radiologists (W-Z. F and J-P. L). Patients were included in our study if they 1) underwent TACE treatment, 2) had an Eastern Cooperative Oncology Group (ECOG) performance status (PS) of 0 or 1, and 3) were Child–Pugh class A or B and had a total bilirubin <34 μmol/L. The survival analysis of these HCC patients was assessed via K–M methods. Our cohort study was approved by the Institutional Review Board of the Research Institute and Hospital National Cancer Center and the institutional review board of The First Affiliated Hospital, Sun Yat-sen University. All patients provided written informed consent.

### Statistical Analysis

Data management and statistical analysis were performed using the R software (version 4.1.0) and GraphPad Prism (version 8.3.0). The Wilcoxon test was used to compare gene expression between the two groups. Differences in proportions were compared by the chi-squared test. Kaplan–Meier curves were plotted, and a log-rank test was applied to check for statistical differences between the survival curves. If not specified, a *p* value <0.05 was considered statistically significant, and all *p* values were two tailed.

## Results

### Datasets

**FIGURE 1:**
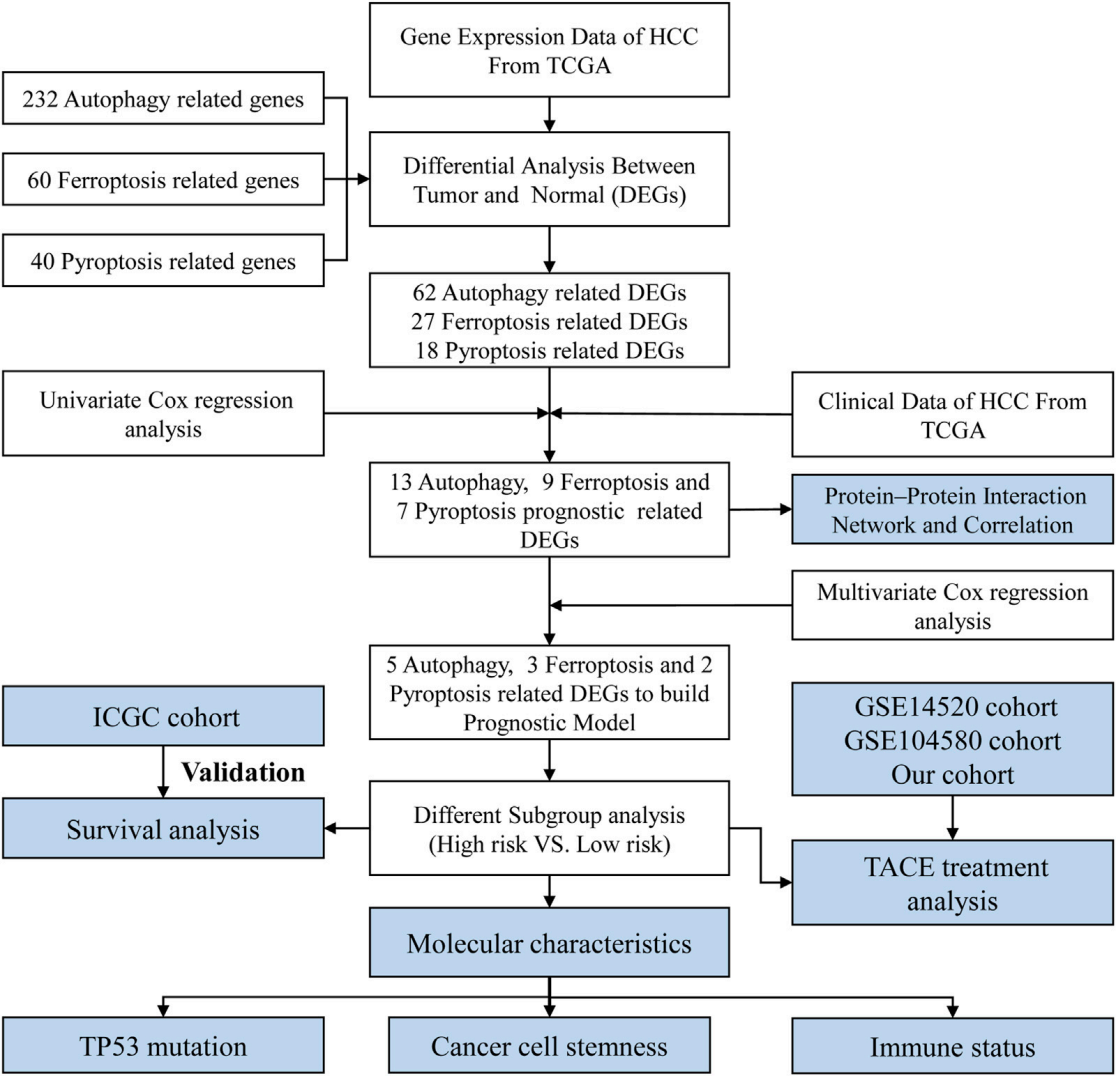
Flow chart of data collection and analysis.

The process for data collection and analysis is depicted in Figure 1. In this study, we included a total of 343 HCC patients from TCGA, 231 HCC patients from ICGC, and 96 HCC patients treated with TACE from GSE14520. The detailed clinical characteristics of these patients are summarized in Supplementary Table S1.

### Identification of Prognostic Autophagy-, Ferroptosis-, and Pyroptosis-Related Genes

Using the R package “limma” for the differentially expressed gene (DEG) analysis, we extracted 62 autophagy-, 27 ferroptosis-, and 18 pyroptosis-related DEGs in the 374 HCC samples and 50 normal liver samples from TCGA. We performed a univariate Cox regression analysis to extract a list of thirteen autophagy-, nine ferroptosis-, and seven pyroptosis-related DEGs that were significantly correlated with OS and found that all of these prognostic DEGs were high-risk factors for poor prognosis in HCC patients (Supplementary Figures S1A–C). PPI network and correlation heatmap and network suggested that there was a potential connection among autophagy-, ferroptosis-, and pyroptosis-related prognostic genes (Supplementary Figures S1D–F).

### Construction and Validation of a Prognostic Model

To determine the independent prognostic genes, multivariate Cox regression analysis for OS was performed among the prognostic thirteen autophagy-, nine ferroptosis-, and seven pyroptosis-related DEGs. Finally, we identified five autophagy-, three ferroptosis-, and two pyroptosis-related DEGs (Supplementary Table S2) to establish a predictive model. The corresponding coefficients and gene expression were used to calculate the risk score. The risk score was calculated as follows: (0.1450955 × expression level of BIRC5 + 0.19642991 × expression level of SQSTM1 + 0.37106235 × expression level of HDAC1 + 0.3770679 × expression level of RHEB +0.34668129 × expression level of ATIC +0.16196511 × expression level of G6PD + 0.4035343 × expression level of ACACA +0.20555184 × expression level of SLC1A5 + 0.28470975 × expression level of BAK1 + 0.44820065 × expression level of GSDME). Risk index = (risk score–min)/(max–min). Following this, the patients in the TCGA, ICGC, and the two GEO datasets were divided into high- and low-risk groups based on median risk index (RI). Interestingly, it was found that the expression of 10 model genes in the high-risk group of four datasets was significantly higher than in the low-risk group (Supplementary Figure S2). Besides this, K–M survival curves based on each of the 10 model genes show that the predicted overall survival time of the low-expression group was significantly longer than that of the high-expression group (*p* < 0.05) (Supplementary Figure S3).

**FIGURE 2:**
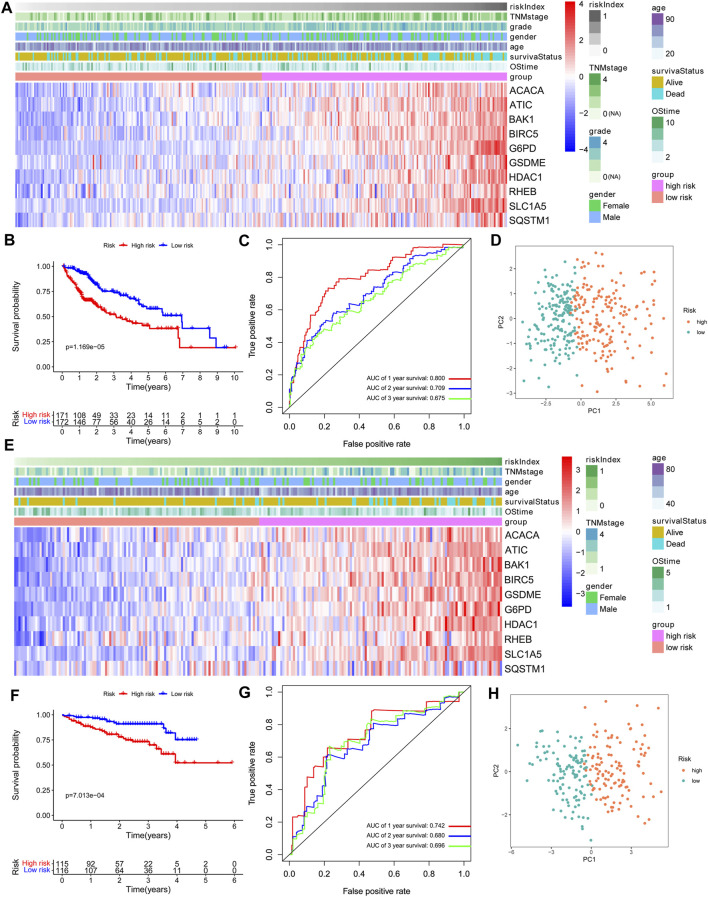
Prognostic value of the 10-gene signature model. **(A,E)** The heatmap of clinicopathological features and 10 model-gene expressions between two subgroups. **(B,F)** Kaplan–Meier curves for OS of patients. **(C,G)** AUC of time-dependent ROC curves. **(D,H)** The results of PCA plot analyses. A–D: TCGA cohort. E–H: ICGC cohort.

From the comparative analysis of patient baseline characteristics of the patients in different risk groups of the TCGA and ICGC cohort, we found that the high-risk group was significantly associated with higher tumor grade and advanced TNM stage in the TCGA or ICGC cohort (Table 1). The relationships among the clinicopathological characteristics, RI, and 10 model-gene expressions were displayed in the form of a heatmap. Patients with higher RI had higher expression of 10 model genes and higher probability of death compared to patients with low RI in the TCGA cohorts (Figure 2A). K–M survival curve outcomes based on median RI values showed that the predicted survival time of the low-risk group, including overall survival (Figure 2B) and disease-free survival (DFS) (Supplementary Figure S4) times, was significantly longer than that of the high-risk group. The predictive performance of RI for OS was evaluated with time-dependent ROC curves. The area under the curve (AUC) reached 0.800 at 1 year, 0.709 at 2 years, and 0.675 at 3 years, respectively (Figure 2C). Additionally, the PCA (Figure 2D) analyses confirmed that HCC patients in different risk groups were distributed in two directions. To test the robustness of the gene signature model constructed from the TCGA cohort, the patients from the ICGC cohort were also categorized into high- and low-risk groups based on the median RI value. The results of the ICGC cohort were similar to the ones from TCGA (Figures 2E–H). In addition, we compared this gene signature with those previously reported PCD-related gene signatures (including autophagy, ferroptosis, and pyroptosis) and found it had displayed comparable or even better in AUCs for OS under certain conditions (Supplementary Table S3).

**TABLE 1:** Baseline characteristics of the patients in different risk groups of the TCGA and ICGC cohort.

Variables	Group	TCGA cohort (n = 343)		*p* value	ICGC cohort (n = 231)		*p* value
		High-risk	Low-risk		High risk	Low risk	
Median survival time (days)		444	672		720	870	
Survival status	Alive	98	126	0.0022	85	104	0.0021
	Dead	73	46		30	12	
Gender	Female	59	51	0.3562	30	31	>0.9999
	Male	112	121		85	85	
Age	≤60	88	77	0.2352	28	21	0.2636
	>60	83	95		87	95	
Grade	G1+G2	83	131	<0.001	—	—	
	G3+G4	85	39		—	—	
	Unknown	3	2		—	—	
Race	Asian	67	66	0.9120	—	—	
	Others	104	106		—	—	
TNM stage	I + II	108	130	0.0137	57	84	<0.001
	III + IV	53	30		58	32	
	Unknown	10	12		0	0	
Hepatitis B/C status	Yes	59	69	0.0827	—	—	
	No	71	78		—	—	
	Unknown	41	25		—	—	

### Independent Prognostic Role of the Gene Signature

**FIGURE 3:**
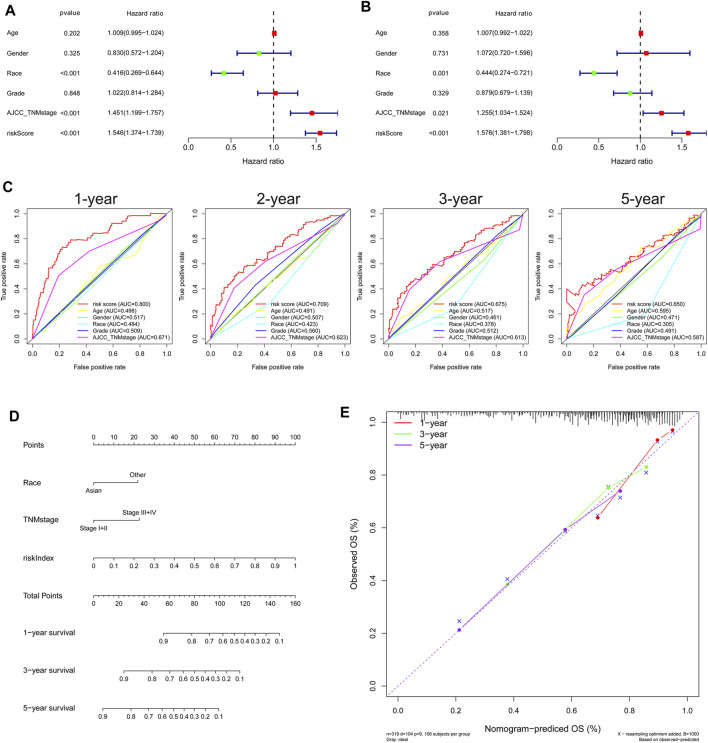
Independent prognostic role of gene signature in the TCGA Cohort. **(A)** Univariate and **(B)** multivariate Cox regression analysis of the associations between risk index (RI) and clinical parameters and OS. **(C)** ROC curve for comparing the prognostic accuracy of RI, age, gender, race, grade, and TNM stage. **(D)** Nomogram for predicting 1-, 3-, and 5-year survival. **(E)** Calibration curves of nomogram on consistency between predicted and observed 1-, 3-, and 5-year survival.

Both univariate Cox and multivariate Cox regression analyses showed that race, TNM stage, and risk score were significantly associated with the prognosis of HCC in the TCGA cohort (Figures 3A,B). Then, HCC patients were stratified according to TNM stage and race, and the correlation between RI and OS was analyzed. The results showed that RI also had a good prognostic effect on HCC patients under different TNM stages and races, indicating that our gene signature could even distinguish the ones with poor survival among the different TNM stage or race patients (Supplementary Figures S5, S6). ROC curve analysis showed that risk score had better predictive accuracy of prognosis than other clinicopathological factors (Figure 3C). Based on race, TNM stage, and risk index (RI), HCC patients with complete clinical information were selected to develop a prognostic nomogram that can be used as a quantitative analysis tool to predict the survival risk of individual patients (Figure 3D). Notably, the calibration curves of the prognostic nomogram showed good consistency between predictive and actual 1-, 3-, and 5-year survival outcomes in the TCGA cohort (Figure 3E).

### Molecular Characteristics of Different Subgroups

**FIGURE 4:**
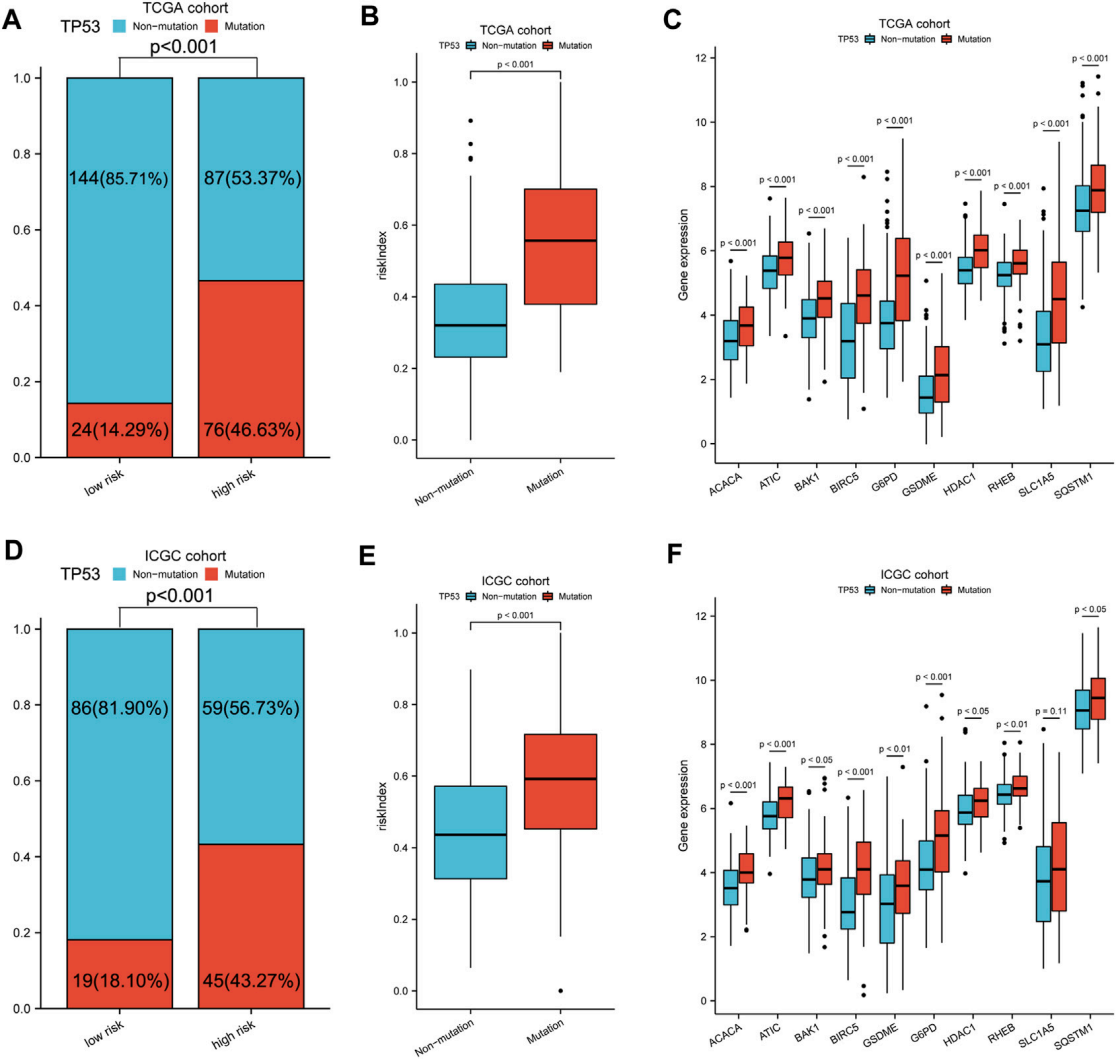
TP53 mutation analysis of different subgroups. **(A,D)** Comparison of TP53 mutation proportion in different subgroups. **(B,E)** Risk index and **(C,F)** 10-model gene expression between TP53 mutation and non-mutation group. A–C: TCGA cohort, D–F: ICGC cohort.

**FIGURE 5:**
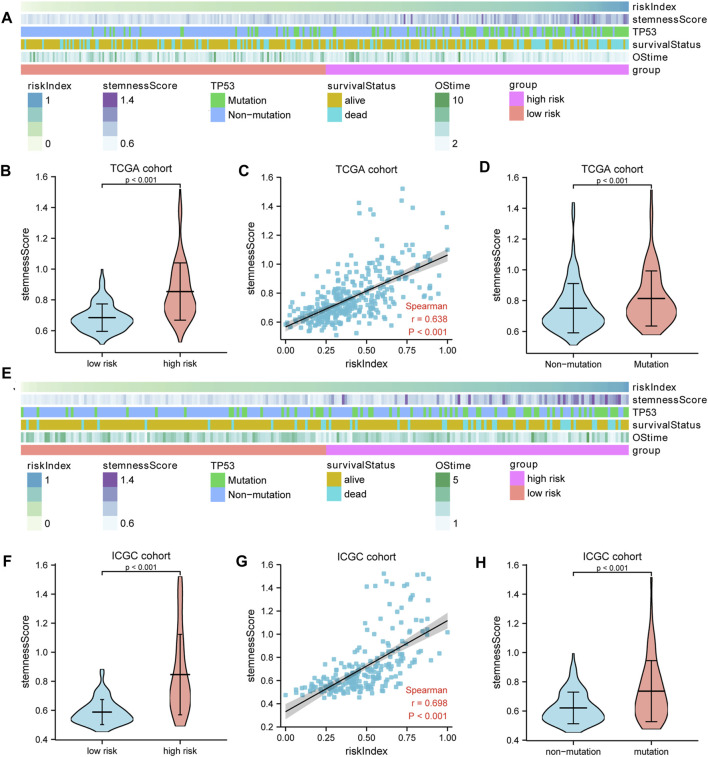
The liver cancer stemness analysis. **(A,E)** The heatmap showed the relationships among risk index, TP53 mutation, stemness score, and survival. The stemness score **(B,F)** in the high- and low-risk groups and **(D,H)** in the TP53 mutation and non-mutation group. **(C,G)** Spearman’s rank correlation analysis between RI and stemness. A–D: TCGA cohort; E–H: ICGC cohort.

TP53 mutation analysis was performed in different subgroups. The high-risk subgroup had a significantly higher TP53 mutation frequency than that of the low-risk subgroup in the TCGA cohort (47 vs. 14%, *p* < 0.001, Figures 4A, Supplementary Figure S7). Furthermore, the expression of these 10 model genes and risk index (RI) in the TP53 mutation group were significantly higher than those of the non-mutation group (Figures 4B,C). Results from the ICGC cohort were similar to those from TCGA (Figures 4D–F). Moreover, ssGSEA was performed to determine the relationship between risk index and liver cancer cell stemness score based on the reported “LCSCs_UP and LCSCs_DN” gene sets (Yamashita et al., 2009). Interestingly, we found that the high-risk or mutation group had a significantly higher stemness score than the low-risk or non-mutation group and had significant positive correlations between RI and stemness (*r* = 0.638, *p* < 0.001) in the TCGA cohort (Figures 5A–D). Similar results were observed in the ICGC cohort (Figures 5E–H).

### Tumor Microenvironment and Immune Response Analysis

**FIGURE 6:**
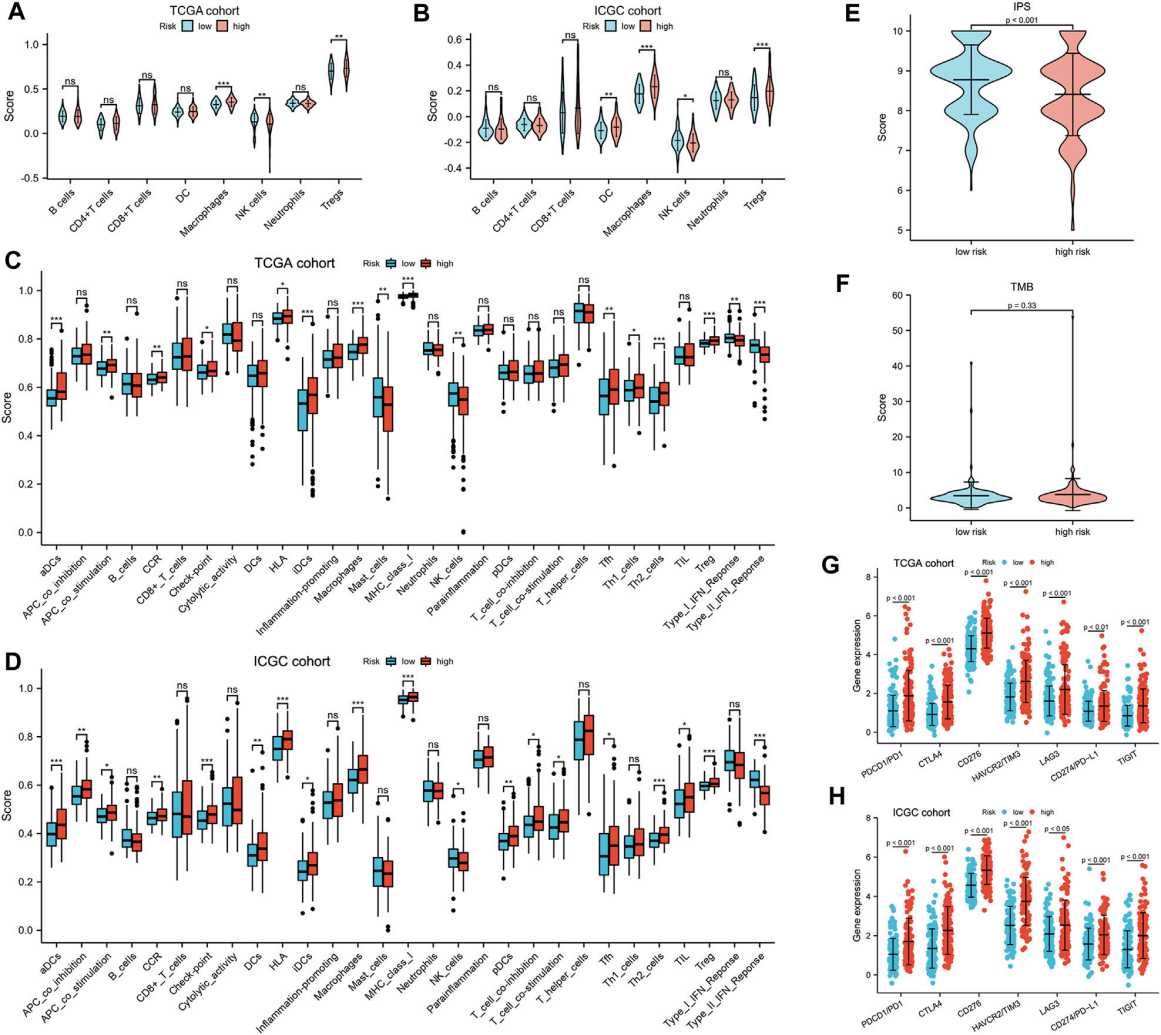
Tumor immune microenvironment analysis. **(A,B)** The eight types of immune infiltration cells in different risk groups. **(C,D)** The ssGSEA scores of 16 immune cells and 13 immune-related functions between the high- and low-risk group. Adjusted *p* values were shown as ns, not significant; *, *p* < 0.05; **, *p* < 0.01; and ***, *p* < 0.001. The **(E)** IPS and **(F)** TMB in different risk subgroups. **(G,H)** The expression of CTLA-4, LAG-3, PD-1, TIGIT, TIM-3, CD276, and PD-L1 in different risk subgroups.

xCell was adopted for evaluation of the relative proportion of the main types of immune infiltration cells. Patients in the high-risk group exhibited a higher level of macrophages and Treg cells, while low-risk patients showed a higher percentage of NK cells (Figures 6A,B). To further explore the tumor immune landscape of high- and low-risk groups, the enrichment scores of different immune cell subpopulations, related functions, and pathways were quantified by ssGSEA. Interestingly, the activity of CCR (cytokine–cytokine receptor), check-point, the scores of macrophages and Treg cells in the high-risk group were higher than those of low-risk patients, while the activity of type I and II IFN response and the scores of NK cells were just the opposite (Figures 6C,D). The results of comparisons in the ICGC cohort were similar to those in the TCGA. The Immunophenoscore (IPS) of the low-risk group was significantly higher than that of the high-risk group, but there was no difference in TMB score (Figures 6E,F). In addition, it was found that the expression of immunosuppressive genes in the high-risk group was higher than that of the low-risk group in both TCGA (Figure 6G) and ICGC cohort (Figure 6H).

### Transarterial Chemoembolization Response Predictive Role of the Gene Signature

**FIGURE 7:**
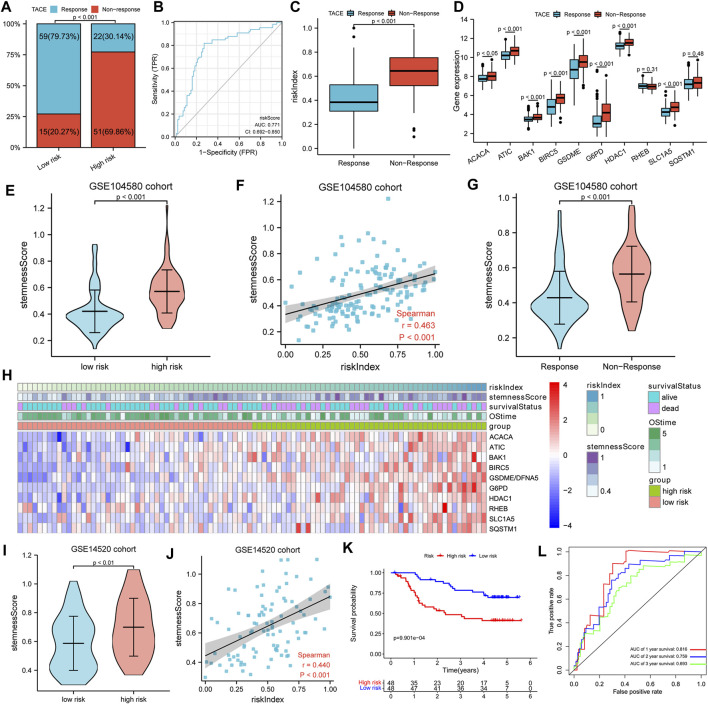
TACE response analysis. **(A)** Comparison of TACE resistance frequency in high- and low-risk groups. **(B)** ROC curves for the predictive performance of RI for TACE responsiveness. **(C,D)** The risk index and gene expression between TACE responsive and non-responsive groups. **(E,G)** The stemness score in different subgroups. **(F)** Spearman’s rank correlation analysis between RI and stemness. **(H)** The heatmap showed the relationships among risk index, stemness score, 10-model gene expression, and survival. **(I)** The Comparison of stemness score in high- and low-risk groups. **(J)** Spearman’s rank correlation analysis between RI and stemness. **(K)** K–M curves for OS of patients. (L) AUC of time-dependent ROC curves.

A total of 147 patients of the GSE104580 cohort were divided into high- and low-risk groups according to the median RI. It was found that the TACE resistance frequency of the high-risk group was significantly higher than that of the low-risk group (70 vs. 20%, *p* < 0.001) (Figure 7A). The predictive performance of RI for TACE responsiveness was evaluated with diagnosis-dependent ROC curves. The area under the curve (AUC) reached 0.771 (95% CI: 0.692–0.850) (Figure 7B). Significantly upregulated BIRC5, HDAC1, ATIC, G6PD, ACACA, SLC1A5, BAK1, and GSDME expressions as well as elevated RI were also observed in the non-responsive group; however, RHEB and SQSTM1 showed no difference between the non-responsive and responsive groups (Figures 7C,D). Moreover, the high-risk or TACE non-responsive group had a significantly higher stemness score than the low-risk or responsive group. Significant positive correlations between the RI and stemness were observed in the GSE104580 cohort (*r* = 0.463, *p* < 0.001) (Figures 7E–G).

To further verify the prognostic value of the gene signature for HCC patients treated with TACE, a total of 96 HCC patients in the GSE14520 cohort treated with TACE were divided into high- and low-risk groups according to the median RI. Detailed clinicopathological characteristics of 96 patients in the GSE14520 cohort were equally distributed between the two risk groups (Table 2). Similarly, we also found that the high-risk group had a significantly higher stemness score than the low-risk group and there were significant positive correlations between the RI and stemness (*r* = 0.440, *p* < 0.001) in the GSE14520 cohort (Figures 7H–J). The survival analysis showed that the survival time of the low-risk group was significantly longer than that of the high-risk group, and AUC values in the ROC curves reached 0.816 at 1 year, 0.759 at 2 years, and 0.693 at 3 years (Figures 7K,L).

**TABLE 2:** Baseline characteristics of the patients in the GSE14520 cohort.

Variables	Group	GSE14520 cohort (n = 96)		*p* value
		High risk	Low risk	
Median survival time (days)		697	1638	
Survival status	Alive	22	34	0.0222
	Dead	26	14	
Gender	Female	3	5	0.7145
	Male	45	43	
Age	≤60	43	43	1
	>60	5	5	
TNM stage	I	17	25	0.1583
	II	16	15	
	III	15	8	
	IV	0	0	
BCLC stage	0	6	2	0.1151
	A	28	37	
	B	4	5	
	C	10	4	
AFP	<300 ng/ml	23	28	0.4135
	≥300 ng/ml	25	20	
Hepatitis B/C status	Yes	46	46	1
	No	2	2	
ALT	≤50 U/L	25	28	0.6817
	>50 U/L	23	20	
Main tumor size	≤5 cm	25	32	0.2122
	>5 cm	23	16	
Multinodular	Yes	8	10	0.7944
	No	40	38	
Cirrhosis	Yes	45	41	0.3167
	No	3	7	

### Verification of the Relationships Among the Gene Signature, TP53 Mutation, Stemness, Immune Status, and Transarterial Chemoembolization Treatment in Our Cohort

**FIGURE 8:**
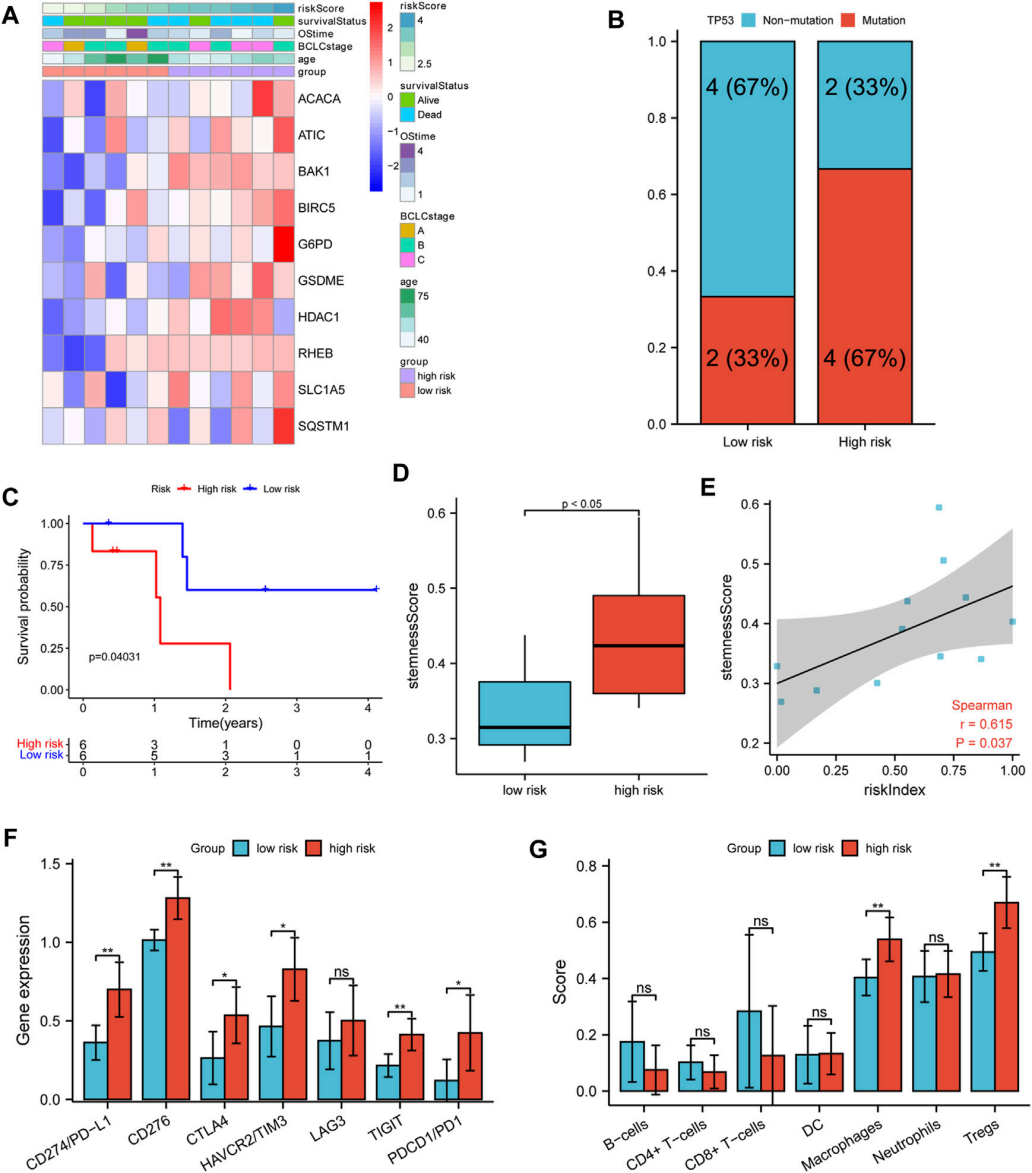
Verifying analysis in our cohort. **(A)** The heatmap of clinicopathological features and 10 model-gene expressions in two risk subgroups. **(B)** The TP53 mutation rate in different risk subgroups. **(C)** K–M curves for OS of patients. **(D)** The stemness score in different risk subgroups. **(E)** Spearman’s rank correlation analysis between RI and stemness score. **(F)** The comparison expression of CTLA-4, LAG-3, PD-1, TIGIT, TIM-3, CD276, and PD-L1 between the high- and low-risk group. **(G)** The immune infiltration cell in different risk groups.

A list of 12 patients with transcriptome sequencing and whole exon sequencing data were divided into high- and low-risk groups according to the median RI. The heatmap indicated that patients with a higher RI had elevated expression of 10 model genes and an increased probability of early death compared to patients with low risk in our cohort (Figure 8A). Additionally, we also found that the TP53 mutation frequency in the high-risk group was higher than that of the low-risk group (Figure 8B). Similarly, the survival analysis suggested that the OS of the low-risk group was significantly longer than that of the high-risk group (Figure 8C). Furthermore, the results also suggested that the high-risk group had a significantly higher stemness score than the low-risk group and that there were significant positive correlations between RI and stemness (*r* = 0.615, *p* < 0.05) (Figures 8D,E). Moreover, it was found that the expression of immunosuppressive genes in the high-risk group was higher than in the low-risk group (Figure 8F), and patients in the high-risk group exhibited a higher level of macrophages and Treg cells, while low-risk patients showed a higher percentage of NK cells by xCell analysis (Figure 8G).

## Discussion

With the beginning of precision medicine and the development of next-generation sequencing and microarray technology, the current treatment and prognosis of HCC have been improved. However, due to the small number of useful biomarkers, it is still challenging to predict the therapeutic effect of its treatment and prognosis in HCC patients. The importance of predicting the prognosis of HCC and administering treatments in a timely manner highlights the need to identify robust prognostic and predictive risk-stratification biomarkers for HCC therapy.

In previous studies, it has been found that non-apoptotic programmed cell death, including autophagy, ferroptosis, and pyroptosis, plays a critical role in the initiation and progression of liver cancer (Hage et al., 2019; Huang et al., 2018; Nie et al., 2018). However, whether these PCD-related genes are correlated with HCC patient prognosis remains largely unknown. In our study, we attempted to develop this novel prognostic model by integrating five autophagy-, three ferroptosis-, and two pyroptosis-related DEGs (BIRC5, SQSTM1, HDAC1, RHEB, ATIC, G6PD, ACACA, SLC1A5, BAK1, and GSDME), which were all upregulated in HCC tumor tissues and associated with poor prognosis (Supplementary Figures S2, S3). Previous studies have confirmed that the five autophagy-related genes (BIRC5, SQSTM1, HDAC1, RHEB, and ATIC) are associated with tumor proliferation, apoptosis, and resistance to anticancer agents in HCC patients. The regulation of HDAC1 and BIRC5 expression could affect the proliferation of HCC cells and induce cell cycle arrest (Zhang et al., 2019; Zhang et al., 2021). The overexpression of ATIC and RHEB was associated with metastasis and poor prognosis in hepatocellular carcinoma (Li et al., 2017; Liu et al., 2018). Tetsuya et al. have reported that inhibiting phosphorylated SQSTM1/p62 may inhibit cell proliferation (Saito et al., 2016). The three ferroptosis-related genes, including lipid the metabolism gene (ACACA) and energy metabolism genes (G6PD, SLC1A5), were also upregulated and correlated with poor prognosis in HCC patients (Lu et al., 2018; Lally et al., 2019; Liang et al., 2020). Additionally, knockdown of pyroptosis-related gene BAK1 expression could inhibit HCC cell proliferation and promote tumor cell apoptosis (Zhu et al., 2020). Wang et al., (2017) also found that GSDME could switch from a caspase-3-mediated apoptosis induced by TNF or chemotherapy drugs to pyroptosis. In our study, the K–M curve and ROC curve indicated that our gene signature had good prognostic performance with high sensitivity and specificity; it was additionally validated in an external cohort. Using our model, we found that the high-risk group was significantly correlated with shorter OS and DFS periods (Figure 2 and Supplementary Figure S4). Independent prognostic analysis additionally showed that risk index (RI) was an independent predictor of survival (Figure 3), suggesting the potential application of our gene signature as a prognostic factor in the clinic. Compared with constructing independently for each PCD-related group of genes, our prognostic model based on integrating all three non-apoptotic programmed cell death-related genes showed profound ability in predicting HCC patients’ prognosis (Supplementary Table S3).

To gain further biological insight into the molecular characteristic, we then studied TP53 mutation and stemness analysis of different subgroups. The TP53 mutation was more common in the high-risk group than in the low-risk group in both the TCGA and ICGC cohorts (Figure 4 and Supplementary Figure S7). The TP53 mutation is not only the single most common genetic event but is also associated with more aggressive disease and worse prognosis in many cancers. In this study, we also found that the expression of the 10 model genes in the TP53 mutant sample was significantly higher. Our gene signature is closely related to TP53 mutation, and TP53 mutation may lead to elevated expression of these genes, affecting the survival prognosis of patients. The stemness analysis by ssGSEA indicated that the stemness of the high-risk or mutation group in the TCGA and ICGC cohorts was all significantly higher than that of the low-risk or non-mutation group. The data also showed significant positive correlations between the risk index and stemness score (Figure 5). Overall, our gene signature is closely related to stemness and the TP53 mutation, which causes poorer outcomes for patients by promoting the proliferation of tumor cells. It is also reasonable to assume that the stemness of the high-risk or TP53 mutation group is elevated, which contributes to the group’s poor prognosis. Collectively, these data indicate that the prognostic gene signature can predict liver cancer stemness and TP53 mutation occurrence and potentially be used to guide HCC treatment decisions.

As an indispensable part of immunotherapy, the tumor immune microenvironment (TIME), whose analysis would help to improve the reactivity of immunotherapy and the potential of precision therapy, has gradually attracted people’s attention (Binnewies et al., 2018). So, we explored the relationship between the risk index and known predictive biomarkers for the TIME and found that the high-risk group had higher fractions of macrophages and Treg cells, but lower fractions of NK cells. Previous studies have demonstrated that the increase of tumor-associated macrophages and Treg cells was associated with poor prognosis in patients with HCC (Zhou et al., 2016). In addition, the impairment of the activity of type I and II IFN response and increased activities of Tfh cells, Treg cells, and macrophage cells in the high-risk group would play an important role in tumor immunological escape and tolerance (Lin et al., 2021). Moreover, we found that the gene expression of immune checkpoints in the high-risk group was higher than that in the low-risk group, but the IPS was lower (Figure 6). So, it is reasonable to assume that the anti-tumor immunity of the high-risk group is attenuated, which may be an important reason for its poor prognosis. Therefore, the prognostic model has the potential to guide immunotherapy decisions.

Current prognostic screening for HCC lacks the ability to identify HCC patients who would benefit from TACE administered, so only some patients exhibit a survival benefit from TACE therapy. So, it is imperative to identify the drivers that promote resistance. Previous studies have reported that the existence of liver cancer stem cells is an important reason for TACE resistance (Wei et al., 2021). Additionally, our previous study suggested that the TP53 mutation is independently related to TACE failure or refractoriness (Xue et al., 2020). The aforementioned analysis indicated that our prognostic gene signature is closely related to the TP53 mutation, which led us to further analyze the relationship between our prognostic model and TACE treatment response. Interestingly, we found that the proportion of TACE resistance in the high-risk group was significantly higher than that in the low-risk group (70 vs. 20%, *p* < 0.001, Figure 7A). Significantly upregulated stemness score, risk index, and most model genes were also observed in the TACE non-responsive group. Moreover, we found that the high-risk group was significantly correlated with shorter OS and higher stemness scores in GSE14520 HCC patients treated with TACE (Figure 7K). Collectively, our gene signature model could predict survival benefits from TACE therapy. Finally, we confirmed the relationships among gene signature, TP53 mutation, stemness, immune status, and the efficacy of TACE treatment in our cohort (Figure 8). In accordance with expectation, the survival analysis suggested that the OS of the low-risk group was significantly longer than that of the high-risk in our institution sequencing data and that the TP53 mutation frequency in the high-risk group was trend higher than that in the low-risk group. In addition, the high-risk group had a significantly higher stemness score than that of the low-risk group. Significant positive correlations between RI and stemness were also observed. Similar to the previous study and public database analysis, the results from our cohort suggest that the anti-tumor immunity of the high-risk group is attenuated. Overall, it is more strongly reasonable to assume that our gene signature model is closely related to TP53 mutation and stemness, tumor immune microenvironment, and the interaction between them affects TACE responsiveness and prognosis of HCC patients.

In summary, a novel prognostic model for HCC that integrates three non-apoptotic programmed cell death-related genes was constructed based on the TCGA databases and validated through the ICGC and GEO datasets as well as our institution sequencing data. Firstly, the prognostic model could accurately predict the prognostic survival status of HCC patients. Secondly, it could estimate the TP53 mutation and liver cancer cell stemness of HCC patients. Thirdly, this model could evaluate the tumor immune microenvironment status of HCC patients. Finally, our gene signature could be used to predict the response and efficacy of HCC patients treated with TACE. Therefore, our gene signature model could provide powerful insights for future treatment options by evaluating the specific conditions of HCC patients.

At the same time, the gene signature model also has its limitations. Firstly, our prognostic model was mostly constructed and validated with retrospective data from public databases. Secondly, the relationships among gene signature, TP53 mutation, stemness, immune infiltration, and TACE therapy have only been confirmed in our small cohort of patients. Thus, it is necessary to perform further prospective analysis to observe the heterogeneity between different populations in large or multicenter cohorts.

## Conclusion

In conclusion, the novel gene signature model based on five autophagy-, three ferroptosis-, and two pyroptosis-related genes, which is a promising prognostic biomarker, was developed and cross-cohort validated. The gene signature may help in distinguishing TP53 mutation, liver cancer cell stemness, immune infiltration, and TACE therapy sensitivity of hepatocellular carcinoma and predicting the outcome of patients. The specific potential mechanism between non-apoptotic programmed cell death-related genes and TP53 mutation, liver cancer cell stemness, tumor microenvironment, or TACE therapy sensitivity in HCC remains unclear, which is worthy of further study.

## Data Availability

Publicly available datasets were analyzed in this study. The data of hepatocellular carcinoma patients in this study were mainly from The Cancer Genome Atlas (TCGA, https://portal.gdc.cancer.gov/), International Cancer Genome Consortium (ICGC, https://dcc.icgc.org/), and Gene Expression Omnibus (GEO, https://www.ncbi.nlm.nih.gov/geo/).

## References

[B1] Al Mamun A. Wu Y. Jia C. Munir F. Sathy K. J. Sarker T. (2020). Role of Pyroptosis in Liver Diseases. Int. Immunopharmacology 84, 106489. 10.1016/j.intimp.2020.106489 32304992

[B2] Bedoui S. Herold M. J. Strasser A. (2020). Emerging Connectivity of Programmed Cell Death Pathways and its Physiological Implications. Nat. Rev. Mol. Cel Biol. 21, 678–695. 10.1038/s41580-020-0270-8 32873928

[B3] Binnewies M. Roberts E. W. Kersten K. Chan V. Fearon D. F. Merad M. (2018). Understanding the Tumor Immune Microenvironment (TIME) for Effective Therapy. Nat. Med. 24, 541–550. 10.1038/s41591-018-0014-x 29686425 PMC5998822

[B4] Cancer Genome Atlas Research Network (2017). Comprehensive and Integrative Genomic Characterization of Hepatocellular Carcinoma. Cell 169, 1327–1341.e23. 10.1016/j.cell.2017.05.046 28622513 PMC5680778

[B5] Chang Y. Jeong S. W. Young Jang J. Jae Kim Y. (2020). Recent Updates of Transarterial Chemoembolilzation in Hepatocellular Carcinoma. Int. J. Mol. Sci. 21, 8165. 10.3390/ijms21218165 PMC7662786 33142892

[B6] Dixon S. J. Lemberg K. M. Lamprecht M. R. Skouta R. Zaitsev E. M. Gleason C. E. (2012). Ferroptosis: an Iron-dependent Form of Nonapoptotic Cell Death. Cell 149, 1060–1072. 10.1016/j.cell.2012.03.042 22632970 PMC3367386

[B7] European Association for the Study of the Liver (2018). EASL Clinical Practice Guidelines: Management of Hepatocellular Carcinoma. J. Hepatol. 69, 182–236. 10.1016/j.jhep.2018.03.019 29628281

[B8] Famularo S. Di Sandro S. Giani A. Lauterio A. Sandini M. De Carlis R. (2018). Recurrence Patterns after Anatomic or Parenchyma-Sparing Liver Resection for Hepatocarcinoma in a Western Population of Cirrhotic Patients. Ann. Surg. Oncol. 25, 3974–3981. 10.1245/s10434-018-6730-0 30244421

[B9] Fang Y. Tian S. Pan Y. Li W. Wang Q. Tang Y. (2020). Pyroptosis: A New Frontier in Cancer. Biomed. Pharmacother. 121, 109595. 10.1016/j.biopha.2019.109595 31710896

[B10] Fritsch M. Günther S. D. Schwarzer R. Albert M.-C. Schorn F. Werthenbach J. P. (2019). Caspase-8 Is the Molecular Switch for Apoptosis, Necroptosis and Pyroptosis. Nature 575, 683–687. 10.1038/s41586-019-1770-6 31748744

[B11] Fuchs Y. Steller H. (2011). Programmed Cell Death in Animal Development and Disease. Cell 147, 742–758. 10.1016/j.cell.2011.10.033 22078876 PMC4511103

[B12] Fujiwara N. Friedman S. L. Goossens N. Hoshida Y. (2018). Risk Factors and Prevention of Hepatocellular Carcinoma in the Era of Precision Medicine. J. Hepatol. 68, 526–549. 10.1016/j.jhep.2017.09.016 28989095 PMC5818315

[B13] Gaba R. C. Groth J. V. Parvinian A. Guzman G. Casadaban L. C. (2015). Gene Expression in Hepatocellular Carcinoma: Pilot Study of Potential Transarterial Chemoembolization Response Biomarkers. J. Vasc. Interv. Radiol. 26, 723–732. 10.1016/j.jvir.2014.12.610 25724086

[B14] Hage C. Hoves S. Strauss L. Bissinger S. Prinz Y. Pöschinger T. (2019). Sorafenib Induces Pyroptosis in Macrophages and Triggers Natural Killer Cell-Mediated Cytotoxicity against Hepatocellular Carcinoma. Hepatology 70, 1280–1297. 10.1002/hep.30666 31002440

[B15] Huang F. Wang B.-R. Wang Y.-G. (2018). Role of Autophagy in Tumorigenesis, Metastasis, Targeted Therapy and Drug Resistance of Hepatocellular Carcinoma. Wjg 24, 4643–4651. 10.3748/wjg.v24.i41.4643 30416312 PMC6224467

[B16] Huo X. Qi J. Huang K. Bu S. Yao W. Chen Y. (2020). Identification of an Autophagy-Related Gene Signature that Can Improve Prognosis of Hepatocellular Carcinoma Patients. BMC Cancer 20, 771. 10.1186/s12885-020-07277-3 32807131 PMC7433127

[B17] Iñarrairaegui M. Melero I. Sangro B. (2018). Immunotherapy of Hepatocellular Carcinoma: Facts and Hopes. Clin. Cancer Res. 24, 1518–1524. 10.1158/1078-0432.ccr-17-0289 29138342

[B18] Ju A. Tang J. Chen S. Fu Y. Luo Y. (2021). Pyroptosis-Related Gene Signatures Can Robustly Diagnose Skin Cutaneous Melanoma and Predict the Prognosis. Front. Oncol. 11, 709077. 10.3389/fonc.2021.709077 34327145 PMC8313829

[B19] Kudo M. (2011). Adjuvant Therapy after Curative Treatment for Hepatocellular Carcinoma. Oncology 81 (Suppl. 1), 50–55. 10.1159/000333259 22212936

[B20] Lally J. S. V. Ghoshal S. Deperalta D. K. Moaven O. Wei L. Masia R. (2019). Inhibition of Acetyl-CoA Carboxylase by Phosphorylation or the Inhibitor ND-654 Suppresses Lipogenesis and Hepatocellular Carcinoma. Cel Metab. 29, 174–182.e5. 10.1016/j.cmet.2018.08.020 PMC6643297 30244972

[B21] Levine B. Kroemer G. (2019). Biological Functions of Autophagy Genes: A Disease Perspective. Cell 176, 11–42. 10.1016/j.cell.2018.09.048 30633901 PMC6347410

[B22] Li M. Jin C. Xu M. Zhou L. Li D. Yin Y. (2017). Bifunctional Enzyme ATIC Promotes Propagation of Hepatocellular Carcinoma by Regulating AMPK-mTOR-S6 K1 Signaling. Cell Commun. Signal 15, 52. 10.1186/s12964-017-0208-8 29246230 PMC5732395

[B23] Liang J.-y. Wang D.-s. Lin H.-c. Chen X.-x. Yang H. Zheng Y. (2020). A Novel Ferroptosis-Related Gene Signature for Overall Survival Prediction in Patients with Hepatocellular Carcinoma. Int. J. Biol. Sci. 16, 2430–2441. 10.7150/ijbs.45050 32760210 PMC7378635

[B24] Lin Z. Xu Q. Miao D. Yu F. (2021). An Inflammatory Response-Related Gene Signature Can Impact the Immune Status and Predict the Prognosis of Hepatocellular Carcinoma. Front. Oncol. 11, 644416. 10.3389/fonc.2021.644416 33828988 PMC8019928

[B25] Liu F. Pan Z. Zhang J. Ni J. Wang C. Wang Z. (2018). Overexpression of RHEB Is Associated with Metastasis and Poor Prognosis in Hepatocellular Carcinoma. Oncol. Lett. 15, 3838–3845. 10.3892/ol.2018.7759 29467900 PMC5796355

[B26] Llovet J. M. Montal R. Sia D. Finn R. S. (2018). Molecular Therapies and Precision Medicine for Hepatocellular Carcinoma. Nat. Rev. Clin. Oncol. 15, 599–616. 10.1038/s41571-018-0073-4 30061739 PMC12452113

[B27] Lu M. Lu L. Dong Q. Yu G. Chen J. Qin L. (2018). Elevated G6PD Expression Contributes to Migration and Invasion of Hepatocellular Carcinoma Cells by Inducing Epithelial-Mesenchymal Transition. Acta Biochim. Biophys. Sin (Shanghai) 50, 370–380. 10.1093/abbs/gmy009 29471502

[B28] Martin S. P. Fako V. Dang H. Dominguez D. A. Khatib S. Ma L. (2020). PKM2 Inhibition May Reverse Therapeutic Resistance to Transarterial Chemoembolization in Hepatocellular Carcinoma. J. Exp. Clin. Cancer Res. 39, 99. 10.1186/s13046-020-01605-y 32487192 PMC7268641

[B29] Mishra A. P. Salehi B. Sharifi-Rad M. Pezzani R. Kobarfard F. Sharifi-Rad J. (2018). Programmed Cell Death, from a Cancer Perspective: An Overview. Mol. Diagn. Ther. 22, 281–295. 10.1007/s40291-018-0329-9 29560608

[B30] Nie J. Lin B. Zhou M. Wu L. Zheng T. (2018). Role of Ferroptosis in Hepatocellular Carcinoma. J. Cancer Res. Clin. Oncol. 144, 2329–2337. 10.1007/s00432-018-2740-3 30167889 PMC11813439

[B31] Park J. W. Chen M. Colombo M. Roberts L. R. Schwartz M. Chen P. J. (2015). Global Patterns of Hepatocellular Carcinoma Management from Diagnosis to Death: the BRIDGE Study. Liver Int. 35, 2155–2166. 10.1111/liv.12818 25752327 PMC4691343

[B32] Saito T. Ichimura Y. Taguchi K. Suzuki T. Mizushima T. Takagi K. (2016). p62/Sqstm1 Promotes Malignancy of HCV-Positive Hepatocellular Carcinoma through Nrf2-dependent Metabolic Reprogramming. Nat. Commun. 7, 12030. 10.1038/ncomms12030 27345495 PMC4931237

[B33] Sieghart W. Hucke F. Peck-Radosavljevic M. (2015). Transarterial Chemoembolization: Modalities, Indication, and Patient Selection. J. Hepatol. 62, 1187–1195. 10.1016/j.jhep.2015.02.010 25681552

[B34] Sung H. Ferlay J. Siegel R. L. Laversanne M. Soerjomataram I. Jemal A. (2021). Global Cancer Statistics 2020: GLOBOCAN Estimates of Incidence and Mortality Worldwide for 36 Cancers in 185 Countries. CA A. Cancer J. Clin. 71, 209–249. 10.3322/caac.21660 33538338

[B35] Tang D. Kang R. Berghe T. V. Vandenabeele P. Kroemer G. (2019). The Molecular Machinery of Regulated Cell Death. Cel Res. 29, 347–364. 10.1038/s41422-019-0164-5 PMC6796845 30948788

[B36] Tang D. Chen X. Kang R. Kroemer G. (2021). Ferroptosis: Molecular Mechanisms and Health Implications. Cell Res. 31, 107–125. 10.1038/s41422-020-00441-1 33268902 PMC8026611

[B37] Toshima T. Shirabe K. Matsumoto Y. Yoshiya S. Ikegami T. Yoshizumi T. (2014). Autophagy Enhances Hepatocellular Carcinoma Progression by Activation of Mitochondrial β-oxidation. J. Gastroenterol. 49, 907–916. 10.1007/s00535-013-0835-9 23702609

[B38] Wang Y. Gao W. Shi X. Ding J. Liu W. He H. (2017). Chemotherapy Drugs Induce Pyroptosis through Caspase-3 Cleavage of a Gasdermin. Nature 547, 99–103. 10.1038/nature22393 28459430

[B39] Wang Z. Ren Z. Chen Y. Hu J. Yang G. Yu L. (2018). Adjuvant Transarterial Chemoembolization for HBV-Related Hepatocellular Carcinoma after Resection: A Randomized Controlled Study. Clin. Cancer Res. 24, 2074–2081. 10.1158/1078-0432.ccr-17-2899 29420221

[B40] Wei X. Zhao L. Ren R. Ji F. Xue S. Zhang J. (2021). MiR‐125b Loss Activated HIF1α/pAKT Loop, Leading to Transarterial Chemoembolization Resistance in Hepatocellular Carcinoma. Hepatology 73, 1381–1398. 10.1002/hep.31448 32609900 PMC9258000

[B41] Xia X. Wang X. Cheng Z. Qin W. Lei L. Jiang J. (2019). The Role of Pyroptosis in Cancer: Pro-cancer or Pro-"host"? Cell Death Dis. 10, 650. 10.1038/s41419-019-1883-8 31501419 PMC6733901

[B42] Xue M. Wu Y. Fan W. Guo J. Wei J. Wang H. (2020). Prognostic Value of TP53 Mutation for Transcatheter Arterial Chemoembolization Failure/Refractoriness in HBV-Related Advanced Hepatocellular Carcinoma. Cancer Res. Treat. 52, 925–937. 10.4143/crt.2019.533 32229792 PMC7373860

[B43] Yamashita T. Ji J. Budhu A. Forgues M. Yang W. Wang H. Y. (2009). EpCAM-positive Hepatocellular Carcinoma Cells Are Tumor-Initiating Cells with Stem/progenitor Cell Features. Gastroenterology 136, 1012–1024. 10.1053/j.gastro.2008.12.004 19150350 PMC2828822

[B44] Ye Y. Dai Q. Qi H. (2021). A Novel Defined Pyroptosis-Related Gene Signature for Predicting the Prognosis of Ovarian Cancer. Cell Death Discov. 7, 71. 10.1038/s41420-021-00451-x 33828074 PMC8026591

[B45] Yu S. Wang Y. Jing L. Claret F. X. Li Q. Tian T. (2017). Autophagy in the "Inflammation-Carcinogenesis" Pathway of Liver and HCC Immunotherapy. Cancer Lett. 411, 82–89. 10.1016/j.canlet.2017.09.049 28987386

[B46] Zhang Y. Chen J. Wu S.-S. Lv M.-J. Yu Y.-S. Tang Z.-H. (2019). HOXA10 Knockdown Inhibits Proliferation, Induces Cell Cycle Arrest and Apoptosis in Hepatocellular Carcinoma Cells through HDAC1. Cmar 11, 7065–7076. 10.2147/cmar.s199239 PMC6666378 31440094

[B47] Zhang M. Yan X. Wen P. Bai W. Zhang Q. (2021). CircANKRD52 Promotes the Tumorigenesis of Hepatocellular Carcinoma by Sponging miR-497-5p and Upregulating BIRC5 Expression. Cel Transpl. 30, 9636897211008874. 10.1177/09636897211008874 PMC8058805 33845641

[B48] Zhou S.-L. Zhou Z.-J. Hu Z.-Q. Huang X.-W. Wang Z. Chen E.-B. (2016). Tumor-Associated Neutrophils Recruit Macrophages and T-Regulatory Cells to Promote Progression of Hepatocellular Carcinoma and Resistance to Sorafenib. Gastroenterology 150, 1646–1658.e17. 10.1053/j.gastro.2016.02.040 26924089

[B49] Zhu J. Tang B. Lv X. Meng M. Weng Q. Zhang N. (2020). Identifying Apoptosis-Related Transcriptomic Aberrations and Revealing Clinical Relevance as Diagnostic and Prognostic Biomarker in Hepatocellular Carcinoma. Front. Oncol. 10, 519180. 10.3389/fonc.2020.519180 33680905 PMC7931692

