# Supplementary material for: Non-Apoptotic Programmed Cell Death-Related Gene Signature Correlates With Stemness and Immune Status and Predicts the Responsiveness of Transarterial Chemoembolization in Hepatocellular Carcinoma
Source: Front Cell Dev Biol. 2022 Apr 29;10:844013. doi: 10.3389/fcell.2022.844013 (PMC9099410; doi:10.3389/fcell.2022.844013)
Supplement: Supplementary file 1 [file DataSheet1.DOCX]

**Non-Apoptotic Programmed Cell Death-related Gene Signature Correlates with Stemness and Immune Status and Predicts the Responsiveness of Transarterial Chemoembolization in Hepatocellular Carcinoma**

**Supplementary Figures**


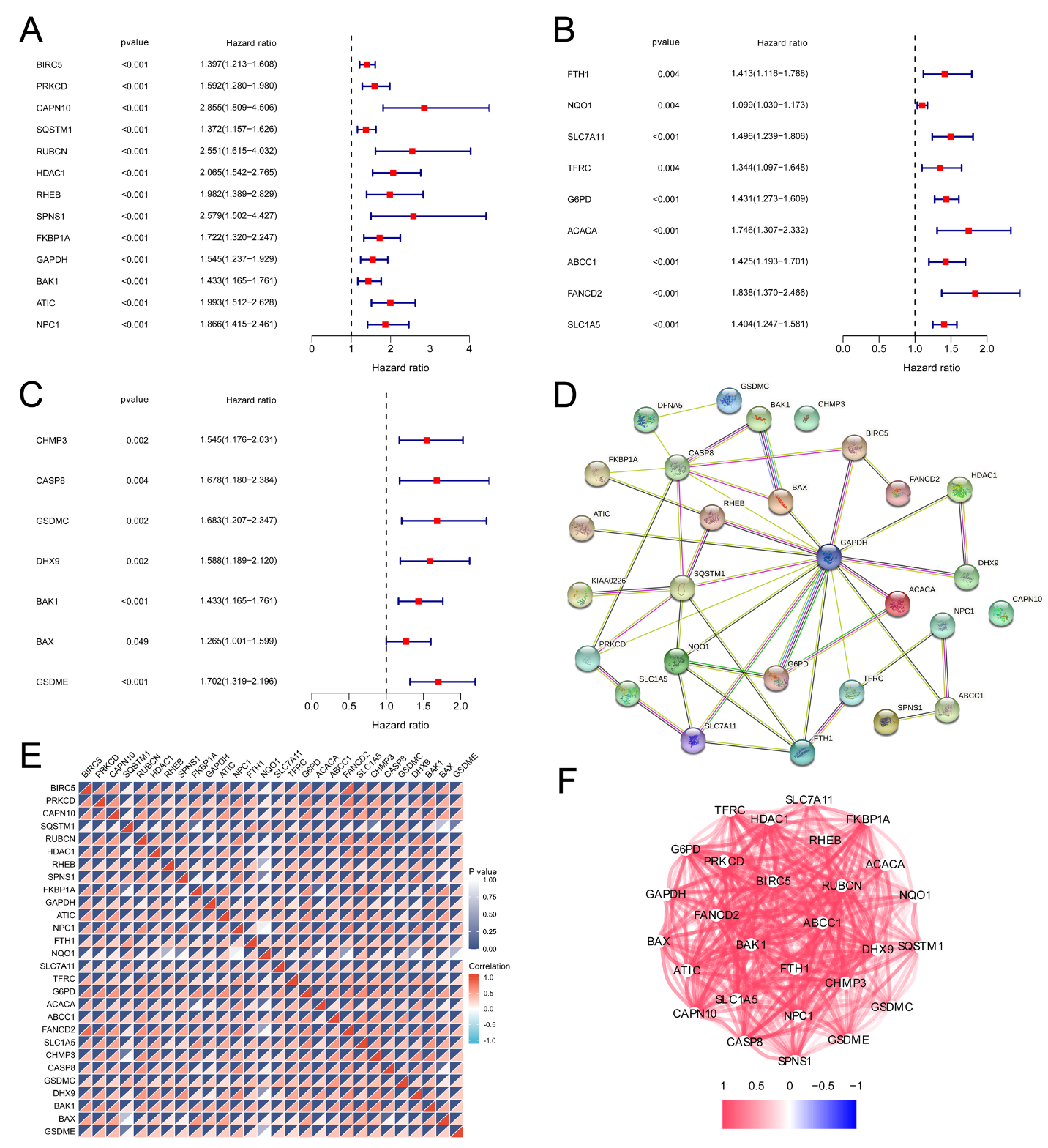


**Figure S1. Identiﬁcation of the candidate genes related to the survival of HCC in TCGA.** Forest plots showing that a list of (A) 13 Autophagy-, (B) 9 Ferroptosis- and (C) 7 Pyroptosis-related DEGs were signiﬁcantly correlated with OS. (D) The protein–protein interaction (PPI) network, (E) correlation heatmap and (F) correlated regulation network of prognostic Autophagy-, Ferroptosis- and Pyroptosis-related DEGs. Data were analyzed using Spearman’s rank correlation analysis.


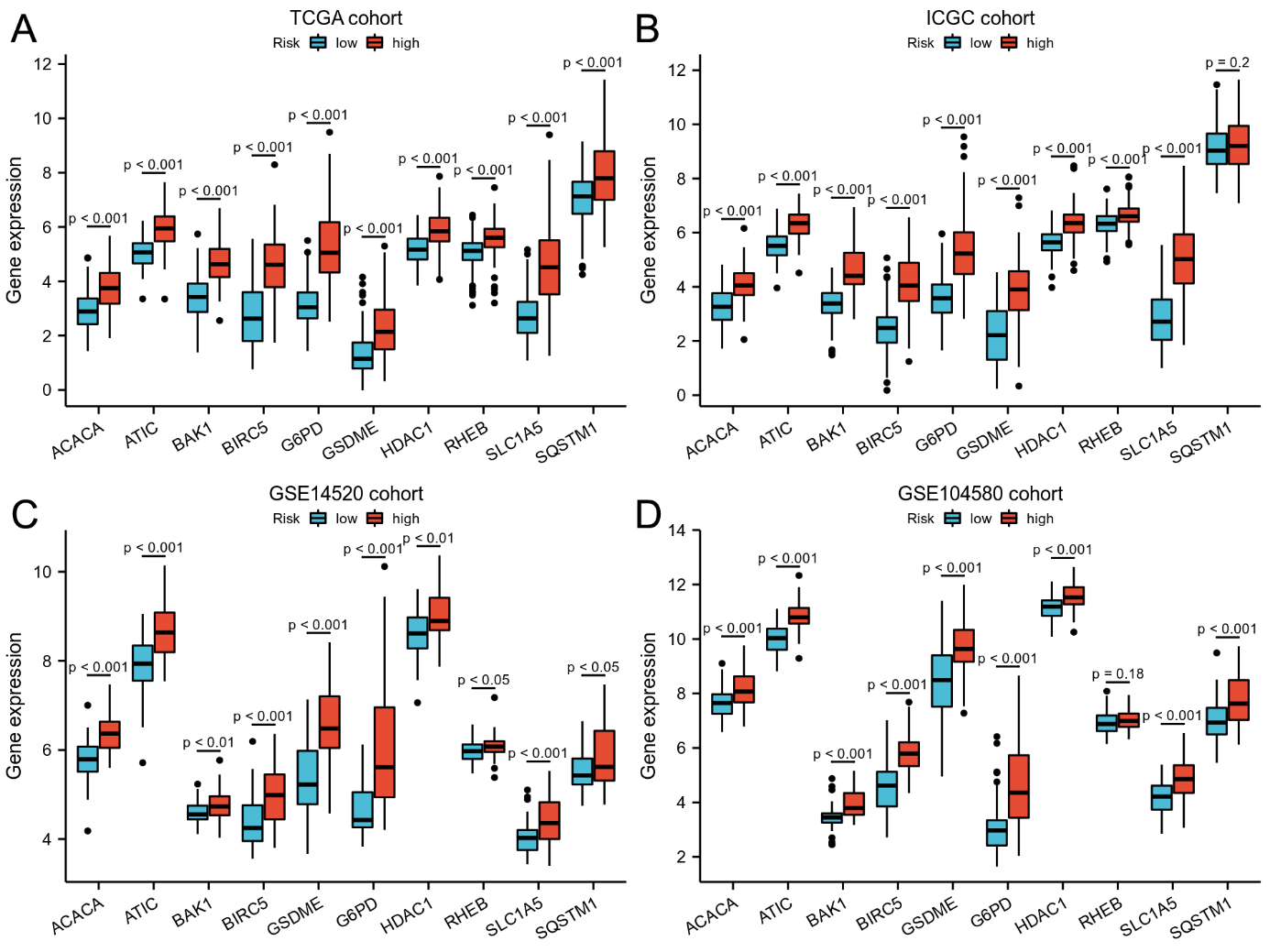


**Figure S2. The expression of 10 model genes in different subgroups.**


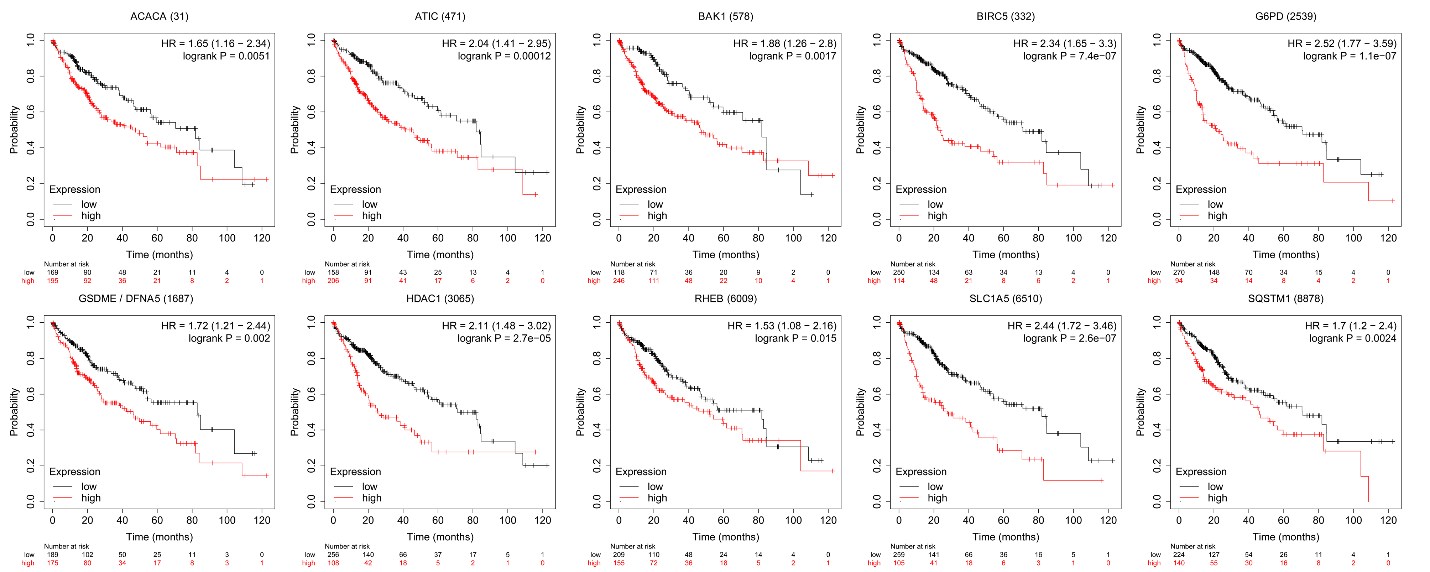


**Figure S3. Kaplan-Meier survival curves of OS based on each of 10 model genes** (accessed from http://kmplot.com/analysis/index.php?p=background)**.**


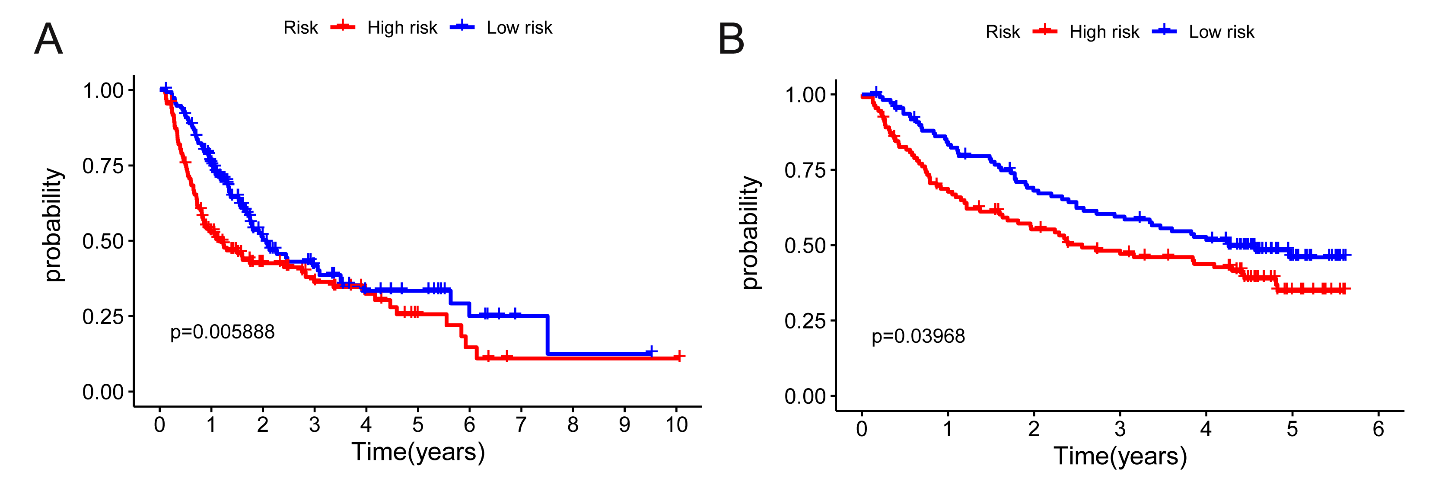


**Figure S4. Kaplan-Meier curves for the disease free survival (DFS) between the high- and low-risk group.** (A) TCGA and (B) GSE14520 cohort.


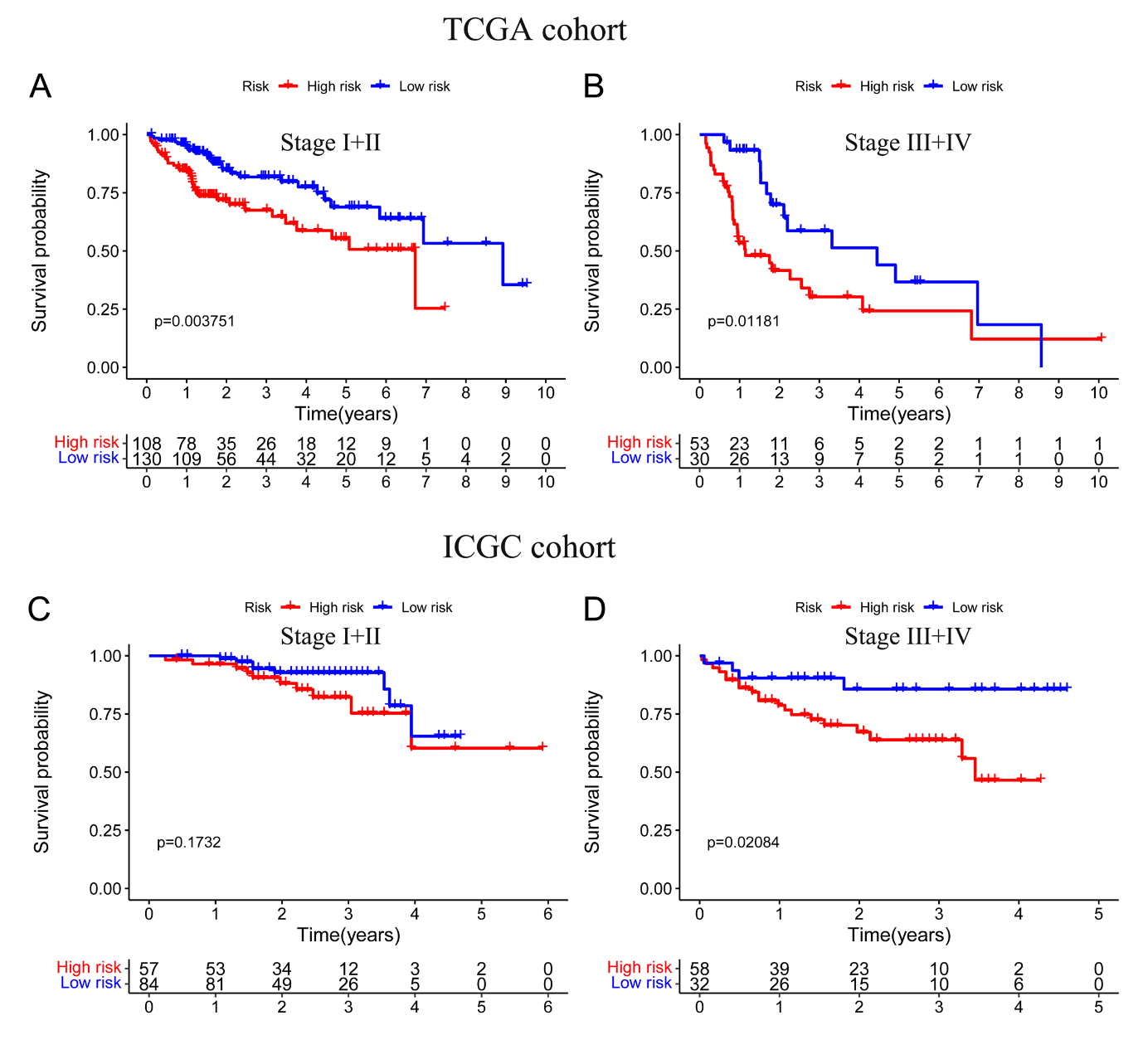


**Figure S5. Kaplan-Meier curves for OS in different tumor stages.**

**
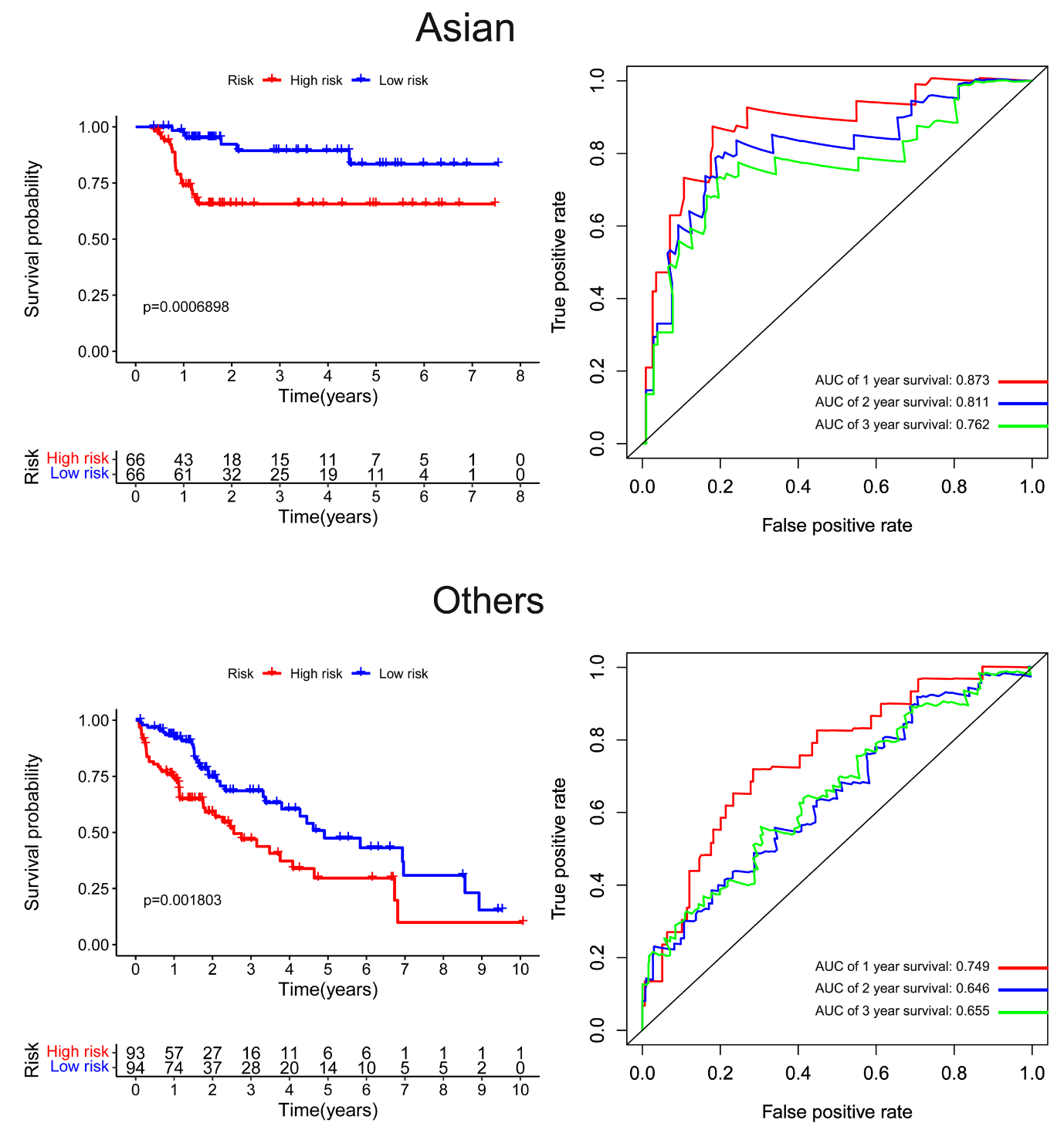
**

**Figure S6. Survival analysis in different Race.**

**
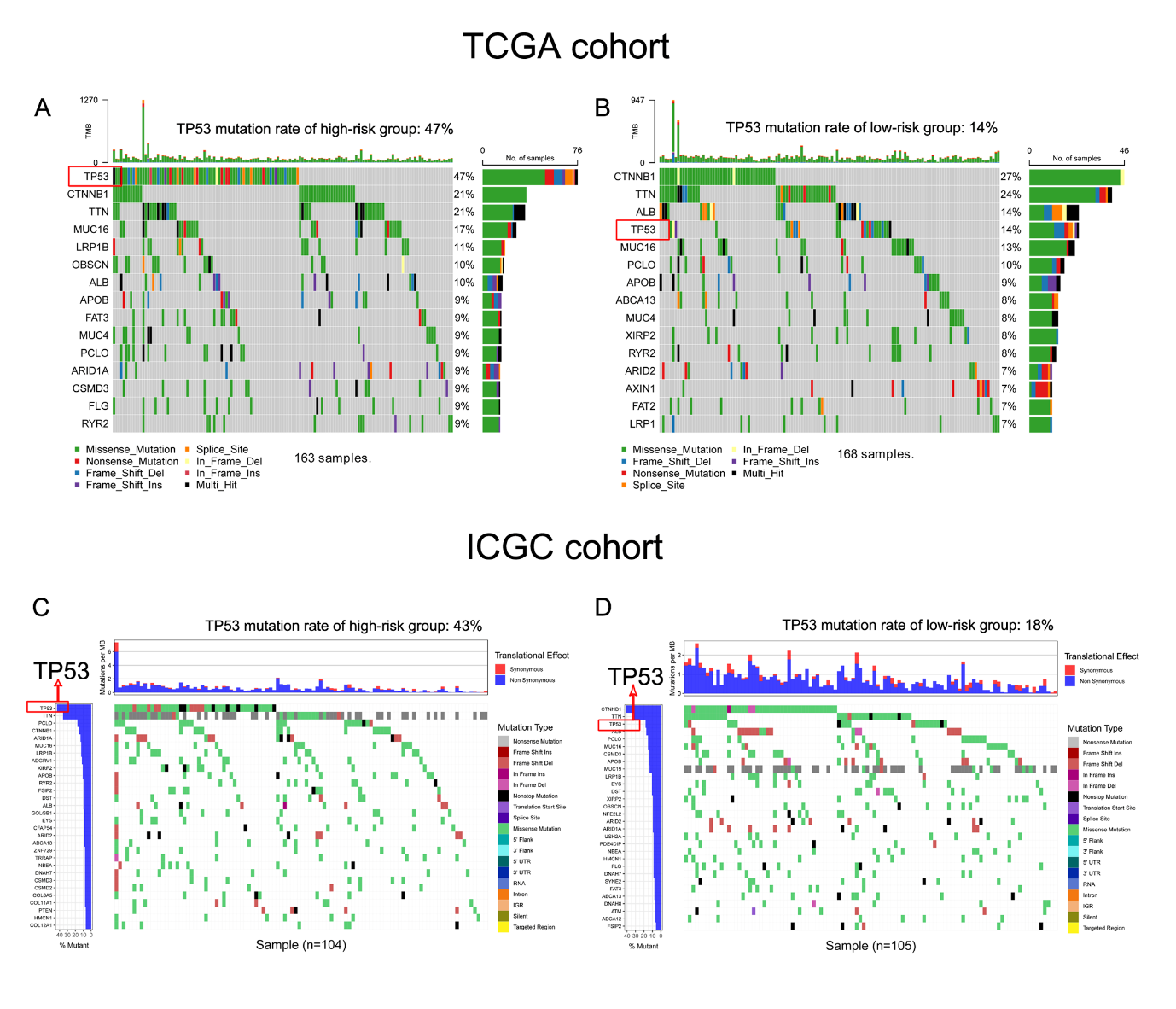
**

**Figure S7. Mutation analysis of different subgroups.** Significantly mutated genes of (A, C) high- and (B, D) low-risk subgroup HCC samples. Mutated genes (rows, top 15) are ordered by mutation rate (TP53 is marked with a red rectangle); samples (columns) are arranged to emphasize mutual exclusivity among mutations. The right shows mutation percentage, and the top shows the overall number of mutations. The color-coding indicates mutation type.

**Supplementary Tables**

Table S1. Patient demographics and clinical characteristics of the included datasets.

| Variables | | Group | TCGA cohort  (n = 343) | ICGC cohort  (n=231) | GSE14520 cohort  (n = 96) |
| --- | --- | --- | --- | --- | --- |
| Median survival time (days) | |  | 587 | 780 | 1534 |
| Survival status | | Alive | 224 (65%) | 189 (82%) | 56 (58%) |
|  | | Dead | 119 (35%) | 42 (18%) | 40 (42%) |
| Gender | | Female | 110 (32%) | 61 (26%) | 8 (8%) |
|  | | Male | 233 (68%) | 170 (74%) | 88 (92%) |
| Age | | ≤60 | 165 (48%) | 49 (21%) | 86 (90%) |
|  | | >60 | 178 (52%) | 182 (79%） | 10 (10%) |
| Tumor grade | | G1 | 53 (15%) | / | / |
|  | | G2 | 161 (47%) | / | / |
|  | G3 | 112 (33%) | / | / |  |
|  | | G4 | 12 (3.5%) | / | / |
|  | | Unknown | 5 (1.5%) | / | / |
| TNM staging | | I | 161 (47%) | 36 (16%) | 42 (44%) |
|  | | II | 77 (23%) | 105 (45%) | 31 (32%) |
|  | | III | 80 (23%) | 71 (31%) | 23 (24%) |
|  | | IV | 3 (1%) | 19 (8%) | / |
|  | | Unknown | 22 (6%) | / | / |

Table S2. Multivariate Cox regression analysis of ten gene signature.

| **gene** | coefficients | HR (95% CI) | P value |
| --- | --- | --- | --- |
| BIRC5 | 0.145096 | 1.16 (0.96-1.39) | 0.117046 |
| SQSTM1 | 0.19643 | 1.22 (1.02-1.45) | 0.027032 |
| HDAC1 | 0.371062 | 1.45 (1.03-2.03) | 0.031487 |
| RHEB | 0.377068 | 1.46 (0.996-2.14) | 0.052691 |
| ATIC | 0.346681 | 1.41 (1.00-1.99) | 0.046986 |
| G6PD | 0.161965 | 1.18 (0.99-1.40) | 0.067597 |
| ACACA | 0.403534 | 1.50 (1.09-2.06) | 0.013471 |
| SLC1A5 | 0.205552 | 1.23 (1.04-1.45) | 0.015339 |
| BAK1 | 0.28471 | 1.33 (1.07-1.65) | 0.008921 |
| GSDME | 0.448201 | 1.57 (1.20-2.04) | 0.000823 |

Table S3. Comparison with previously reported prognostic models

| Ref. | Model | Training set | AUC  (1-, 3-, 5-year OS) or (1-, 2-, 3-year OS) | Validation set one | AUC  (1-, 3-, 5-year OS) or (1-, 2-, 3-year OS) | Validation set two | AUC  (1-, 3-, 5-year OS) or (1-, 2-, 3-year OS) |
| --- | --- | --- | --- | --- | --- | --- | --- |
| Our model | 10-gene | TCGA(343) | 0.800, 0.709, 0.675 | ICGC(231) | 0.742, 0.680, 0.696 | GSE14520(96) | 0.816, 0.759, 0.693 |
| Wenfang Xu *et al*.^[1]^ | 8-gene | TCGA(365) | 0.732, 0.701, 0.656 | GSE14520(221) | 0.656, 0.637, 0.606 | GSE36376(223) | 0.71, 0.666, 0.614 |
| Jieying Liang *et al* ^[2]^ | 10-gene | TCGA(365) | 0.800, 0.690, 0.668 | ICGC(231) | 0.680, 0.690, 0.718 | / | / |
| Jianfeng Ding *et al*.^[3]^ | 11-gene | TCGA(370) | 0.785, 0.710, 0.671 | ICGC(231) | 0.750, 0.772, 0.503 | / | / |
| Shuang Liu *et al*.^[4]^ | 6-gene | GSE14520(221) | 0.724, 0.774, 0.678 | GSE76427(115) | 0.597, 0.691, 0.762 | / | / |
| Shuqiao Zhang *et al*.^[5]^ | 7-gene | TCGA(345) | 0.753, 0.616, 0.639 | ICGC(231) | 0.663, 0.642, 0.638 | / | / |
| Min Deng *et al*.^[6]^ | 10-gene | TCGA(358) | 0.770, 0.713, 0.693 | GSE10186(118) | 0.641, 0.663, 0.681 | / | / |

**Reference**

[1] XU W, GUO W, LU P, et al. Identification of an autophagy-related gene signature predicting overall survival for hepatocellular carcinoma [J]. Biosci Rep, 2021, 41(1).

[2] LIANG J Y, WANG D S, LIN H C, et al. A Novel Ferroptosis-related Gene Signature for Overall Survival Prediction in Patients with Hepatocellular Carcinoma [J]. Int J Biol Sci, 2020, 16(13): 2430-41.

[3] DING J, HE X, LUO W, et al. Development and Validation of a Pyroptosis-Related Signature for Predicting Prognosis in Hepatocellular Carcinoma [J]. Frontiers in genetics, 2022, 13: 801419.

[4] LIU S, SHAO R, BU X, et al. Identification of the Pyroptosis-Related Gene Signature for Overall Survival Prediction in Patients With Hepatocellular Carcinoma [J]. Front Cell Dev Biol, 2021, 9: 742994.

[5] ZHANG S, LI X, ZHANG X, et al. The Pyroptosis-Related Gene Signature Predicts the Prognosis of Hepatocellular Carcinoma [J]. Front Mol Biosci, 2021, 8: 781427.

[6] DENG M, SUN S, ZHAO R, et al. The pyroptosis-related gene signature predicts prognosis and indicates immune activity in hepatocellular carcinoma [J]. Mol Med, 2022, 28(1): 16.
